# Brain Correlates of Eating Disorders in Response to Food Visual Stimuli: A Systematic Narrative Review of FMRI Studies

**DOI:** 10.3390/brainsci13030465

**Published:** 2023-03-09

**Authors:** Alessia Celeghin, Sara Palermo, Rebecca Giampaolo, Giulia Di Fini, Gabriella Gandino, Cristina Civilotti

**Affiliations:** 1 Department of Psychology, University of Turin, 10124 Turin, Italy; 2 Neuroradiology Unit, Department of Diagnostic and Technology, Fondazione IRCCS Istituto Neurologico Carlo Besta, 20133 Milan, Italy; 3 Faculty of Educational Science, Salesian University Institute (IUSTO), 10155 Turin, Italy

**Keywords:** anorexia nervosa, bulimia nervosa, binge eating disorder, fMRI, systematic narrative review

## Abstract

This article summarizes the results of studies in which functional magnetic resonance imaging (fMRI) was performed to investigate the neurofunctional activations involved in processing visual stimuli from food in individuals with anorexia nervosa (AN), bulimia nervosa (BN) and binge eating disorder (BED). A systematic review approach based on the PRISMA guidelines was used. Three databases—Scopus, PubMed and Web of Science (WoS)—were searched for brain correlates of each eating disorder. From an original pool of 688 articles, 30 articles were included and discussed. The selected studies did not always overlap in terms of research design and observed outcomes, but it was possible to identify some regularities that characterized each eating disorder. As if there were two complementary regulatory strategies, AN seems to be associated with general hyperactivity in brain regions involved in top-down control and emotional areas, such as the amygdala, insula and hypothalamus. The insula and striatum are hyperactive in BN patients and likely involved in abnormalities of impulsivity and emotion regulation. Finally, the temporal cortex and striatum appear to be involved in the neural correlates of BED, linking this condition to use of dissociative strategies and addictive aspects. Although further studies are needed, this review shows that there are specific activation pathways. Therefore, it is necessary to pay special attention to triggers, targets and maintenance processes in order to plan effective therapeutic interventions. Clinical implications are discussed.

## 1. Introduction

Eating disorders (EDs) are so widespread today that their increase is taking on the contours of a true “social epidemic”, as Gordon predicted as early as 1990 [1]. A wealth of scientific, historical and sociocultural evidence underlines the fact that the processes governing eating behavior are extremely complex and multifaceted. It is now clear that these mechanisms have their roots in the phylogenetically oldest areas of the brain, common among different species, and it is also clear that they branch out into the neocortical areas that have specifically developed in humankind, giving rise to an intricate network that links the primal needs associated with survival to the semantic, value-based and exploratory systems of sharing and enjoyment that have some of their highest expressions in ritualization of food intake [2]. Indeed, it is a widely held and shared belief that food can help manage an emotional state by evoking an immediate sense of well-being and relaxation. However, it is equally clear that this associative schema, when applied with rigidity and regularity or in a dysregulated manner, can lead to states of psychophysical decompensation characterized by an inability to recognize and modulate negative emotional states, such as anxiety, sadness, anger and stress [3,4,5,6,7,8]. From this perspective, anorexia nervosa (AN), bulimia nervosa (BN) and binge eating disorder (BED), which are part of the nutrition and eating disorders cluster (DSM-V: APA, 2013) [9], are complex pathologies that affect both mental state and physical functioning [10]. Other disorders described in DSM 5 include pica, rumination disorder, avoidant/restrictive food intake disorder and other specified feeding or eating disorder (OSFED), but these are estimated to occur in a much smaller proportion of the population [10,11,12].

These pathologies are considered “severe” due to their complex and multifactorial etiology, protracted course, tendency to become chronic (20–30% according to DSM-5) and comorbidity with other mental disorders and medical conditions. Specifically, eating disorders frequently co-occur with anxiety disorders (53%), mood disorders (43%), self-injurious behaviors (21%) and substance use disorders (10%) and frequently co-occur with several medical conditions, such as obesity, diabetes and celiac disease. In addition, these disorders have a high risk of suicide that cannot be fully explained by comorbid disorders [10]. Overall, eating disorders significantly increase risk of death and are the second leading cause of death in adolescent girls and the leading cause of death in psychiatric disorders, with a crude mortality rate of 4% [12,13].

The complex system of influences that can contribute to onset of an eating disorder also includes certain personality traits that constantly regulate the person’s interaction with the world and influence their thought patterns, emotions and the connotation of certain emotions as unpleasant. A tendency toward perfectionism, i.e., the habit of demanding high-quality performance from oneself and criticizing oneself disproportionately, is one of the personality traits that have been highlighted by researchers as potential risk factors for occurrence of dysfunctional eating behaviors and which are located in the neocortical areas responsible for semantic processing [14,15,16,17,18]. To this group of traits, Culbert, Racine and Klump [14] added widespread emotional lability, which determines strong impulsivity or a tendency to behave impulsively, especially as a means of coping with strong negative emotions, but which can also involve marked compulsivity or exaggerated effort to control one’s behavior. These features are traditionally associated with dysregulations at the limbic level [17,18,19]. A tendency toward avoidance, excessive sensitivity to meeting others’ expectations and receiving rewards, low levels of extraversion and marked self-determination are also significantly elevated in individuals with ED [14,20]. In terms of functioning, deficits in some neurocognitive processes have also been reported as risk factors in the literature. In particular, deficits in cognitive flexibility, i.e., the ability to switch quickly and easily from one task to the next or from one strategy to the next, and difficulties in inhibitory control, i.e., the ability to suppress automatic responses, seem to characterize the executive functions of individuals who develop an eating disorder. Cognitive flexibility represents an element of vulnerability prior to development of a disorder that exposes the individual to development of maladaptive behaviors related to eating and complicates their remission. Difficulties in cognitive flexibility seem to mainly affect people with anorexia nervosa and bulimia nervosa, whereas a deficit in inhibitory control, even if it does not fully explain their behavior, is more common in disorders characterized by uncontrolled eating and the tendency to eliminate foods [14].

### 1.1. Anorexia Nervosa, Bulimia Nervosa and Binge Eating: Definitions, Symptomatolog and Epidemiology

Anorexia nervosa (AN) is an eating disorder characterized by low weight, food restriction, a disturbed body image, a fear of gaining weight and an overpowering desire to be thin [21]. In 20–30% of cases, it becomes a chronic condition that can persist for many years (and often throughout the life cycle), leading to impairments in interpersonal functioning and educational or vocational careers. People with AN have a mortality rate five to ten times higher than age- and sex-matched controls. Some studies in the literature have reported that AN has the highest mortality rate of all psychiatric disorders in young women [22,23]. As for the prognosis, almost half of the cases are expected to heal, about one third of the cases improve and the remaining fifth become chronic [24]. Bulimia nervosa (BN), on the other hand, is an eating disorder characterized by excessive food intake followed by episodes aimed at getting rid of the ingested amount of food via methods such as self-induced vomiting or use of laxatives, both at least once a week for 3 months [9]. To date, the first distinction between a person suffering from AN (the subtype with binge eating and elimination behaviors) and a person suffering from BN is usually body weight: in the first case, a body mass index (BMI) well below normal values is usually recorded, whereas bulimic individuals are often of normal weight. Regarding incidence rates, up to 3% of females and more than 1% of males suffer from this disorder during their lifetime [25,26], and some studies indicate prevalences in clinical populations of over 10% [27]. Disease onset usually occurs between 12 and 35 years of age, with higher incidence between 18 and 25 years. In males, the disease peaks in late adolescence and early adulthood, with prevalence increasing between 14 and 20 years of age (0.4% at age 14, 0.7% at age 17 and 1.6% at age 20) [28].

BED diagnosis is characterized by the frequency of binge eating episodes, at least 1 per week over a 3-month period, and originally sparked clinical interest because it was associated with obesity [27,29]. With an incidence of 3.5% in women and 2% in men, the disease generally begins at an older age than BN and AN, around the age of twenty [27,30,31], and it represents the most common diagnosis in male subjects [30]. Regarding the ratio between males and females, epidemiological studies report more homogeneous results than other EDs (from 1:2 to 1:6) [32].

### 1.2. Cerebral Response to Visual Stimuli of Food in Healthy Subjects

Regulation of eating behavior is characterized by synergistic combination of neural activity from numerous regions of the central and peripheral nervous systems. Our literature search revealed reports of neural activity in the anterior cingulate cortex (ACC) [33,34], medial and lateral prefrontal cortex (PFC) [35,36] and orbitofrontal cortex (OFC) [34,37,38,39]. Some areas of the parietal cortex also appear to be activated, such as the postcentral gyrus (PoCG) [40,41], whereas, at the occipital level, greater activation is observed in the fusiform gyrus (FFG) [42] and occipital gyrus (OG) [41,43]. Consistent with the cortical areas is also the activity of the insula (INS) [34,39,44,45] and some subcortical structures, such as the amygdala (AMG) [34,42,46,47] and striatum (STR) [48,49,50]. Activation in the nucleus accumbens (NAc) has also been observed during prediction and after food consumption. The NAc seems to be involved in the cognitive processes of aversion, motivation, reward and reinforcing mechanisms of action [51].

Factors regulating neural response to food stimuli include the salience of a stimulus [52] and evaluation of its reward value [53]. Salience is encoded in the PFC, mainly due to involvement of the right lateral area, OFC and dorsal ACC but also the supplementary motor area (SMA), INS, PoCG and FFG [51,54,55]; in contrast, the medial part of the OFC, rostral ACC and dorsal and ventral part of the STR are involved [51], and the posterior cingulate cortex (PCC) is involved in evaluation of reward value [56,57,58,59].

In summary, control of human appetite appears to occur through two distinct neural circuits: the first, involving the OFC, INS, hypothalamus (HYP), parts of the STR and AMG, would be activated during fasting to promote eating behavior; the second, involving ventromedial and dorsolateral parts of the PFC, would be activated in a state of satiety to stop food intake [60,61,62,63].

The aim of the present work was to examine and discuss, through a systematic search of recent literature, the possible neural correlates involved in processing of visual stimuli with food and neutral content, with a focus on eating disorder pathologies.

## 2. Materials and Methods

The systematic review was conducted according to the PRISMA (Preferred Reporting Items for Systematic Reviews and Meta-Analyses) guidelines for searching, systematizing and reporting systematic reviews [64,65] to achieve the following objectives: (1) to identify the common trends of fMRI studies that have used food visual stimuli to investigate the cerebral activations of AN, BN and BED, and (2) to describe some possible associations with the clinical manifestations to better inform the therapeutic pathway for individuals affected by AN, BN and BED.

### 2.1. Search Strategy

Three electronic databases—Scopus, PubMed and Web of Science (WoS)—were used. The search was conducted through 22 June 2022, with no restrictions on language or time period. We decided not to limit the search to a specific timeframe to maximize the inclusion parameters. Boolean operators were applied to the keywords identified as follows: (image * OR visual) AND (food) AND (fMRI) AND (anorexia OR bulimia OR binge eating OR eating disorder*).

### 2.2. Inclusion Criteria

The inclusion criteria consisted of (a) peer-reviewed original research papers and scientific reports; (b) articles that included selected search terms in the title, abstract and/or keywords; (c) publications in English or Italian (languages spoken by the authors) and (d) articles that presented fMRI results on responses to visual stimuli related to eating in individuals with eating disorders (anorexia nervosa, bulimia nervosa and binge eating disorder). Given the methodological heterogeneity of the included studies, no restrictions were placed on the time factor (i.e., longitudinal vs. cross-sectional; duration of longitudinal study) and/or study design (i.e., randomized control trial, etc.).

### 2.3. Selection of Studies

**Figure 1:**
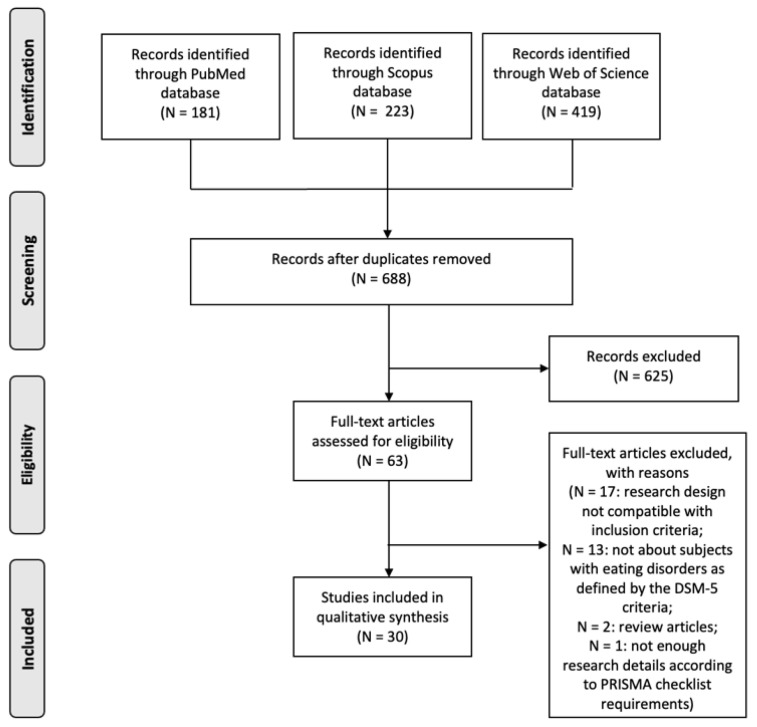
PRISMA flowchart of the study selection process.

Figure 1 shows the selection process according to the PRISMA flowchart. The search strategy yielded a total of 823 relevant records (i.e., 181 in PubMed, 223 in Scopus and 680 in WoS). After removing duplicate entries, N = 688 articles remained. The initial review excluded 625 entries that did not fit the topic. Subsequently, two researchers (C.C. and R.G.) systematically reviewed 63 relevant records and excluded articles that did not meet the criteria. The full texts that were deemed suitable by at least one of the authors were shortlisted. A third researcher (A.C.) mediated disagreements between researchers during the screening process. In the next phase, the eligibility phase, a full-text evaluation of these filtered articles revealed 33 records that did not meet the eligibility criteria described. The following studies were excluded in this phase: N = 17 because the research design did not meet the inclusion criteria; N = 13 because they did not involve individuals with eating disorders as defined by DSM-5 criteria; N = 2 because they were review articles and N = 1 because it contained insufficient research details as per the PRISMA checklist requirements. In total, 30 studies provided empirical evidence that met the described criteria.

For each included study, the following information was extracted: authors, year of publication, eating disorder(s) and the patients’ and healthy controls’ (HC) mean or range age, all of which are reported in Table 1, as well as the research paradigm, stimulus, conditions, duration of the stimulus, fMRI contrast, template, brain area and coordinates, which are reported in the Appendix A.

The extracted data were then summarized in a narrative synthesis organized by each specific eating disorder (AN, BN and BED).

## 3. Results

Ten of the thirty selected articles included only AN patients (five of which compared AN and recovered AN patients and one of which compared younger and older AN patients), three included only BN patients and four included only binge eating patients; three of these presented comparisons between AN and BN and three presented comparisons between BN and BED patients. Sample sizes ranged from a minimum of five to a maximum of 42 subjects, and all but two studies contained a comparison group consisting of healthy controls. The meta-sample thus resulted in 640 subjects, divided by diagnostic class as follows: 406 AN, 128 BN and 106 BED patients.

### 3.1. Anorexia Nervosa

Several studies in the literature have examined the neural responses of AN patients to images of foods compared with neutral/non-food images using fMRI, as shown in the Appendix A.

**Figure 2:**
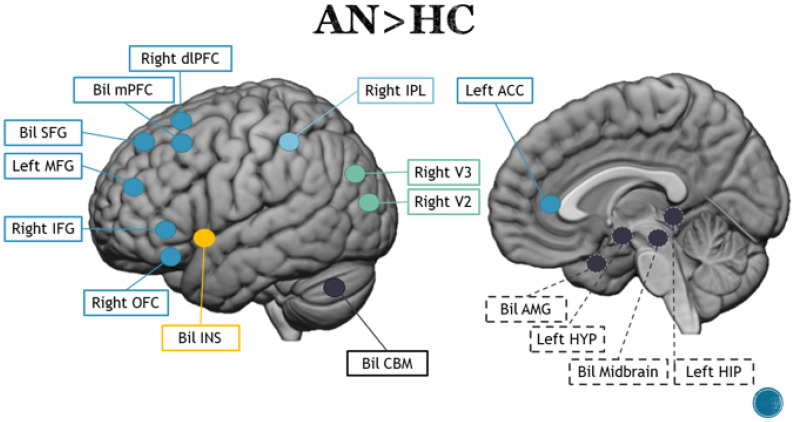
Main brain areas observed in the contrast between anorexic patients and healthy controls. Blue lines represent frontal areas, light blue represent parietal areas, green lines represent occipital areas, yellow lines represent the insula region and black lines represent subcortical regions and the cerebellum. Dotted lines represent mesial regions, whereas solid lines represent lateral and superficial areas.

Presentation of food images to both AN patients and HCs showed that a neural signal was predominantly observed in the INS, OFC and PFC, but, in the AN subjects, there was a decrease in activity in the posterior/medial part of the cingulate cortex and an increase in the right AMG [79], as presented in Figure 2. Whenever subjects saw pictures depicting food and non-food items and were simultaneously asked to think about eating the depicted food, the AN group showed lower cerebellar activation compared to the control group but increased activity in the visual cortex [69]. Increased activity in the posterior visual areas was also confirmed in a study conducted in a group of young (13–18 years) AN patients [95]. When food and non-food conditions were compared, there was greater activation in occipital regions and less activity in temporal and parietal gyri. This comparison, when presenting sweet foods, extended the activation of the occipital regions to the hippocampus. Increased activity in occipital visual areas was also observed when comparing AN patients vs. AN-recovered patients, as demonstrated by Göller and colleagues [76]. A comparison between the two groups (AN and HC) showed that AN had higher BOLD responses compared to HC in the medial cingulate cortex (MCC), precentral gyrus (PrCG), PoCG and parietal areas and no significant group differences for the INS or AMG. A study by Kim and colleagues [81] focused primarily on the role of INS in clinical differentiation of AN and BN. To this end, the authors compared three groups of subjects (AN, BN and controls) during passive visualization of images depicting high-calorie foods and neutral stimuli. The results showed greater activation of the anterior INS in response to food stimuli for both groups when compared to the HC group, but this was correlated with activity of different areas in the two disorders. In comparison to the HC group, the AN group demonstrated greater activity in response to food images in the right inferior frontal gyrus (IFG), superior frontal gyrus (SFG), ACC and cerebellum (CBM) [81].

Satiety plays an important role in weight control [96]; accordingly, studies on eating disorders have investigated how perception of visual food can change as a consequence of fed and fasting conditions. Santel and colleagues [86] compared neural activity of food/non-food visual stimuli in both satiety and hunger states in AN patients and HCs. They detected an increase in neural activity for the AN group in the inferior occipital gyrus (IOG), cerebellum and lingual gyrus (LG) under the satiety condition, whereas they recorded an increase in activation in the cuneus (CUN) and fusiform gyrus (FFG) under the hungry condition. When compared to HCs, AN patients showed reduced activity in the inferior parietal lobe (IPL) in the satiated condition, whereas they showed diminished activation in the right LG when in the hungry condition. Similar fMRI results have been demonstrated previously [90,91] but without reference to hunger or satiety. Lawson and colleagues [82] used the same paradigm by presenting images of food to AN patients and HCs before and after meals. In AN participants, fMRI examination revealed hypoactivity in the HYP, AMG, hippocampus (HIP), OFC and INS in the pre-meal condition and in the AMG and INS in the post-meal condition. A study by Rothemund and colleagues [84] showed that, in a fasting state, compulsive acts, which are typical of AN patients, were correlated with activation of the claustrum during the high-calorie condition and predicted several deactivations of frontal and temporal regions, with the data showing that, in AN patients, this effect was specific to hunger and did not occur in the satiated state [84].

Appearance of visual stimuli related to food can have a considerable impact on one’s motivation to eat [97]. The motivational salience expressed by, for example, high- or low-calorie food images influences the decision to consume or refrain from eating certain foods. Furthermore, the visual qualities of food and other contextual signals can be quickly conditioned as secondary reinforcers, which can then influence future eating-related behavior [98,99,100]. When asking how much of each visual food stimulus the patients wanted to eat, Scaife and colleagues found a pattern of reversed activation of the lateral frontal lobe between AN patients and the control group, specifically an increase in activity for high-calorie foods and a decrease in activity for low-calorie foods in the AN group and the opposite activation pattern in the control group [87]. In addition, the AN patients showed lower activation than the other subjects in the somatosensory regions in response to both visual stimuli. Horndasch and colleagues [78] included a more heterogeneous group of participants and compared two groups of AN patients, one containing adults and one containing adolescents, with their respective control groups. The stimuli consisted of photographs of low-calorie and high-calorie foods, as well as positive, negative and neutral emotional photos. In the comparison between the two groups of adults, AN patients showed greater activation in the cerebellum for both types of food stimuli but a decrease in activity in the IFG and thalamus (THAL) for low-calorie stimuli. In the adolescent groups, AN patients exhibited greater activation in several areas: the IFG, medial PFC and INS for high-calorie stimuli and the left cerebellum, medial PFC and IPL for low-calorie stimuli. In both cases, the control group showed greater activation in the right cerebellum. In the comparison between the two AN groups, adults showed greater activation in the SPL and right cerebellum, whereas adolescents showed greater activation in the ACC, superior frontal lobe (SFL) and left cerebellum.

However, in acute anorexia nervosa, cognitive and physiological systems are severely disturbed and it is not possible to determine whether certain abnormalities are a cause or consequence of starvation [101]. To avoid the confounding effects of current starvation, studies have also investigated neural activity related to perception of visual food stimuli in recovered AN patients. This is especially significant because it is well known that people who have recovered continue to exhibit basic eating disorder symptoms [102]. Uher and colleagues [90,91] compared brain activation during processing of sweet and savory food stimuli and aversive and neutral emotional content in AN patients, recovered AN patients and healthy women as a control group [90]. The recovered female patients showed greater activation at the level of ACC and medial PFC but also a decrease in activity in the inferior parietal lobe (IPL) compared to HCs. In addition, compared with the chronic patients, the recovered women also showed increased activity in the dorsal ACC and PFC, both right lateral and apical. According to the authors, activation of areas common to chronic patients and recovered women, such as the medial PFC and ACC, may represent markers of disease. Low activity in the apical and lateral prefrontal areas may also be considered an indicator of pathology as the response in these areas is observed in recovered subjects and the control group but not in chronic patients. In 2015, Sanders and colleagues [85] found greater activity in the caudate nucleus (CN) in recovered AN patients compared to HCs, as well as in the right cerebellum. The left hippocampus and cerebellum are mainly activated in AN and recovered AN and not in HCs, whereas activation of the HYP has been reported for AN and HCs but not for recovered AN patients. Insular activity was observed only in recovered AN patients and HCs but not in AN patients. At the cortical level, activity in the left medial frontal gyrus (MFG) was observed in HCs but not in AN and recovered AN patients, whereas activity in the right MFG emerged in both AN groups but not in the HC group.

### 3.2. Bulimia Nervosa

**Figure 3:**
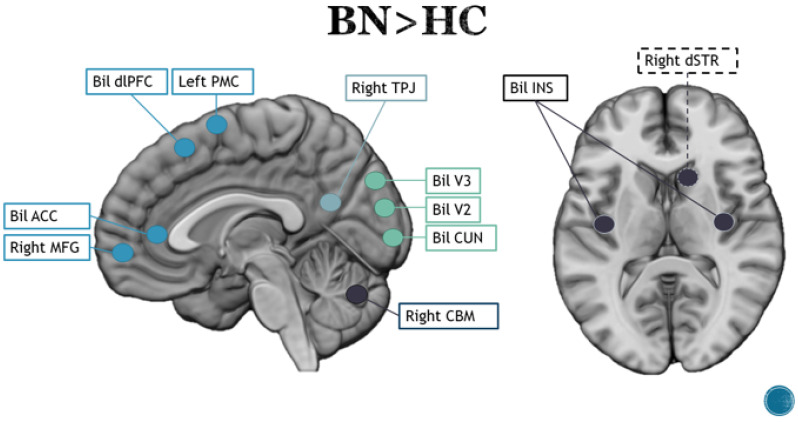
Main brain areas observed in the contrast between bulimic patients and healthy controls. Blue lines represent frontal areas, light blue represent parietal areas, green lines represent occipital areas and black lines represent subcortical regions and the cerebellum. Dotted lines represent mesial regions, whereas solid lines represent lateral and superficial areas.

Similar paradigms to those used for studying AN have been used in subjects with BN, as listed in Appendix A, and authors have often compared the results obtained by studying different eating disorders. In general, individuals with an eating disorder show greater activation of the ACC and right cerebellum in response to food stimuli [91], as shown in Figure 3. Looking at the neural activity of BN patients and HCs during processing of images depicting food, Van de Eynde and colleagues [92] found that both groups showed greater activation of the left MFG and visual areas. In addition, subjects from BN showed greater involvement of the superior frontal gyrus (SFG) and bilateral CUN compared to HCs [92]. Joos and colleagues [80] observed a decrease in general activity, especially in relation to ACC and PFC, in BN patients compared to HCs. This controversial reduction in activity in the ACC, which was not confirmed in other studies, could be explained by the hunger or satiety state in which the subjects were studied [80,103]. When comparing neural activity of BN patients, AN patients and HCs while observing and thinking about visual food stimuli in a state of hunger, in the BN group, there was greater activation of the medial PFC compared to AN patients but less activation of the lateral PFC compared to the control subjects. Furthermore, greater activation of the lateral PFC was found in AN patients compared with the other two groups. As mentioned above, increased activity in the medial PFC and ACC is considered a marker for AN, but it could also be a common feature of eating disorders [91]. Studies using high- and low-calorie stimuli identified specific activation in each condition: the left cerebellum, right STG, right MTG and left caudate in HCs; the right V1, left dlPFC, left INS and left PrCG in BN patients and the left cerebellum, right PFC and right precuneus (preCUN) in AN patients. In the comparison between BN and AN patients, greater activation of the right CN, right STG/INS and left SMA was found in the BN group, as well as increased activity in the right parietal lobe and left posterior cingulate cortex (PCC). The contribution of the INS represented an interesting key aspect in the clinical differentiation of AN and BN [104]. The results showed greater activation of the anterior INS in response to food stimuli for both groups, but this was correlated with activity of different areas in the two disorders. When compared to HCs, the BN group showed increased activity in the right MFG, right INS and cerebellum, whereas, compared to the AN group, BN patients showed increased activity in the right MTG [81]. When having patients focus on a specific emotional sensation, such as foods or objects that elicit disgust, researchers have observed an interesting difference between BN and BED conditions. The results obtained after 12 h of fasting showed greater activation of the ACC and left INS in BN patients compared to the other two groups; furthermore, insula activation was positively associated with a “degree” of uncontrolled eating and negatively associated with blood glucose levels [88]. A recent study examined individual differences in the BOLD response in the appetitive network (AMG, OFC, INS, STR) as moderators of the relationship between craving and binging while testing women with BN in an ecological environment. The authors found that BN subjects exhibited generally significantly increased activation in the left AMG in response to food cues compared to neutral cues [93].

### 3.3. Binge Eating Disorder

**Figure 4:**
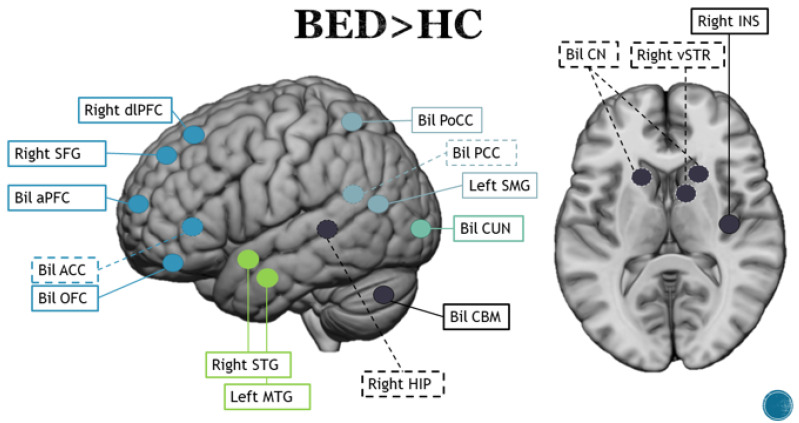
Main brain areas observed in the contrast between binge eating disorder patients and healthy controls. Blue lines represent frontal areas, light blue represent parietal areas, green–blue lines represent occipital areas, green lines represent temporal regions and black lines represent subcortical regions and cerebellum. Dotted lines represent mesial regions, whereas solid lines represent lateral and superficial areas.

The results regarding neural activity of BED patients viewing food and non-food images are listed in Appendix A Significant and are shown in Figure 4. differences in neural activity were found only in the group of obese participants with BED compared to HCs and only when stimuli depicting binge eating (desserts and high-fat salty snacks) were presented: four out of five of them actually showed activation in the ventral part of the premotor cortex (vPMC) [74]. Using high- and low-calorie stimuli and non-food stimuli under two conditions (fed and fasting), Dimitropoulos and colleagues [71] found greater activation in the anterior region of the PFC in the pre-meal condition for both types of food and in the SFG and cerebellum for low-calorie food in the BED group. After a meal, the BED group responded more strongly to images of high-calorie foods in the lateral OFC, ACC, CN, PFC, MFG and HIP. There was greater activation of the anterior and dorsolateral PFC, SFG, temporal lobe, CN and PCC for images of low-calorie foods [71]. Dodds et al. [72] obtained similar results, finding that processing of food images was associated with activation of a network of reward areas, including the AMG, STR and INS. When focusing on high- and low-energy processed food, the BED group was associated with greater blood-oxygenation-level-dependent activity (BOLD) in emotional, motivational and somatosensory brain areas, and images of high-energy processed food versus low-energy unprocessed food resulted in greater activity in inhibitory brain regions [66]. As shown in previous studies, comparisons with HCs benefit from confrontation of different eating disorders, so the aforementioned study by Schienle et al. [88] compared the brain responses of BED, BN and healthy controls to images of high- and low-calorie foods, disgusting objects and neutral objects after a 12 h fast. They found that participants from the BED group responded with greater activation of the lateral and medial OFC to stimuli depicting food compared to participants from BN, whereas they showed greater involvement of the medial OFC compared to the control group. Activation of the lateral area of the OFC seems to be of particular interest because it has been associated with inhibitory control of habitual motor responses. In response to images of food, the BED group also showed greater activation in the ventral STR compared to the BN and HC groups [83]. Regarding activation of different parts of the STR, the ventral region seems to be part of the circuits involved in substance dependence, whereas the dorsal area is more involved in control of actions that lead to rewarding behavior [105]. A recent study suggested that the general decremental neural activation of BED patients when presented with high-energy food stimuli may decrease, suggesting disengagement with foods that may be more consistent with those consumed during a binge eating episode [73].

## 4. Discussion

Eating behavior has commonly been considered a hinge shared by different psychopathologies, and, for this reason, classification of different eating disorders has been merged into the same diagnostic category in clinical manuals, such as the DSM. However, the three disorders differ markedly in terms of their symptomatologic constellation and clinical manifestations, and the neurofunctional correlates that characterize each disorder show activation of different brain areas. A comprehensive picture of the neuroactivities related to each ED could be the key to better understanding the underlying mechanisms in etiopathogenetic and explanatory terms and in planning of effective and targeted therapeutic interventions. Unfortunately, due to methodological incongruency among the different studies, in terms of the experimental designs, tasks and stimuli presented, making comparisons and generalized considerations regarding the cerebral correlates of EDs is particularly complex. Still, in our attempt to characterize each eating disorder, we summarize below the main neural evidence emerging from the selected literature and the clinical implications that bridge the neural correlates with the typical symptomatology of each ED.

In all studies of AN, cortical activity has been consistently described in the PFC, ACC, SFG, MFG, IFG and OFC, whereas a clear trend has not emerged regarding activity in the limbic areas (HYP, AMG, HIP and INS), although it appears that neuronal activity is increased in these areas [79,81]. This finding is consistent with other original research and meta-analytic studies [78,106,107] and also with the clinical observation that patients with AN have a cognitive profile that targets high levels of top-down control, in conjunction with emotional dysregulation that is poorly recognized and difficult to manage [108]. The lateral part of the PFC is specifically human and performs control functions in selection of tasks that drive behavior [109]. This area appears to be involved in high-level processing, regardless of the type of stimulus presented, and plays a key role in decision-making processes. It controls behavioral choices when the reward or perceptual stimulus is not directly related to a predetermined action. Its connections to the dorsolateral PFC could indicate involvement in top-down control processes [110,111]. According to the authors, this activation pattern would correlate with restricted consumption of high-calorie foods, which requires greater control by prefrontal regions [87]. This hyperactivation could also play a role in interoceptive perception and hunger state detection (involving corticolimbic structures such as the AMG) or appetite regulation (involving the hypothalamus). These aspects are extremely impaired in AN patients, probably not only because hunger stimuli are not taken into account at the explicit level but also, especially in the most severe cases, due to a rigid mechanism of semantic top-down control that impairs adequate regulation of food intake and affects the limbic components of the brain [108]. Furthermore, it is interesting to note that top-down activations are mostly related to motivational processes that point at personal meanings and values [112,113,114], which might be reflected in the clinical manifestations of AN, such as perfectionism, low self-esteem, low self-confidence, lack of awareness of emotions and attempts to control them, especially in relation to the restricted subtype [13,115,116,117]. Considered together, the difficulties associated with low self-esteem and constant experience of powerlessness may be related to inability to correctly interpret stimuli and sensations emanating from the limbic system, which entails a need for tight control of all visceral needs, especially hunger [118]. Modulation of attention affects level of activation in the sensory cortex. Intensity of attention to a stimulus correlates directly with strength of activation in the corresponding sensory cortex [119,120]. The current data suggest that patients with AN pay less attention to food stimuli in a state of hunger. In daily life, such attentional mechanisms might help anorectic individuals resist eating and maintain fasting. During the satiety state, such suppression of attention to food may not be necessary, which explains why subjects from AN showed greater occipital activation in the satiety state than in the starvation state. From a clinical perspective, work on bodily and emotional awareness and better management of control strategies seems to indicate that they also induce changes at the cerebral level, both in terms of top-down and bottom-up mechanisms [121,122].

BN shows different symptomatology, associated with a different clinical manifestation, although it sometimes alternates with the rigid top-down control characteristic of AN; it may thus occur in the same patient at different times [123] and is characterized by episodes of dysregulated eating followed by feelings of emptiness and guilt and compensatory behaviors [9]. The symptomatologic proximity of BN to problems related to impulsivity and emotional dysregulation [124,125,126] observed in clinical practice is supported by several fMRI studies (e.g., the studies by Schienle et al. [88] and Wonderlich [93]) showing higher activation of the INS, AMG, FG and STR. All these structures are involved in mediating the motor inhibition process and appear to play a role in impulsive behavior [127,128,129]. Regarding activation of neocortical structures, the BN group was found to exhibit greater activation of the medial PFC compared to the AN group but less activation of the lateral PFC compared to the control subjects. Hyperactivity of the medial region (OCD) [130] and comorbidity between eating disorders and OCD are well documented in the literature [131,132]. Although the two disorders—BN and OCD—appear to be distinct from each other, they share a common pattern involving an initial moment of emotional dysregulation and later strong activation due to feelings of guilt and the need to perform compulsive behaviors. The alternation between the two phases of BN and OCD is configured in both disorders as a mechanism for maintaining symptomatology in a vicious cycle [133,134]. In addition, it appears that BN individuals develop a form of addiction to tempting food [135,136], which may explain the hyperactivity of the medial PFC.

In BN patients, involvement of the insula seems to be of particular importance when considered in the context of emotional dysregulation phenomena as it connects the brainstem to neocortical areas and acts as a kind of “interoceptive cortex” that integrates information from somatic perceptual activity with the emotional, behavioral and motivational parameters that characterize higher cortical levels. Clinical work on emotional regulation, particularly in relation to impulsivity, such as that highlighted by Hail and Le Grange [137], seems best suited to address the disorders related to the neural correlates that have emerged from the studies considered. In contrast to the other two disorders, control does not seem to be properly adopted in any way in BED patients, neither on the restrictive side (as in AN) nor as a means of coping with guilt (as in BN patients). From a clinical point of view, the symptomatology of BED is associated with dissociative and addictive manifestations [138,139]. Frequently, these patients report feeling completely “disconnected” during binge eating episodes, and several studies suggest that dissociation is a key factor in predicting BED and episode severity [140,141,142]. Consistent with these clinical premises, our systematic review found activation in the PFC and temporal cortex in these patients. Other studies have found that these regions are activated during dissociative processes [143,144,145]. Along with dissociative symptomatology, activation of the OFC and PFC may maintain binge eating as recurrent as they are among the areas involved in drug addiction [146]. Activation of the CN in the post-eating state, along with the STR and HIP, is also of interest as a positive correlation has been found between CN activity, particularly its functional connection with the PCC and inhibition of avoidance behaviors [147]. Working on dissociation and addiction through psychotherapy with the aim of establishing integration of the different parts of the ego with BED subjects could be a key factor for successful psychotherapy [148].

### 4.1. Limitations

The partial lack of clear consistency in the evidence of neural activation observed in subjects with ED may be due to heterogeneity in research designs, implementation strategies, contexts and outcomes. First, brain activation areas were examined with different task types and evaluation methods to elicit a brain response to a visual food stimulus, so potential reliability and reproducibility biases should be noted. It is also likely that the studies yielded false-positive results due to small sample sizes, as well as different statistical methods (e.g., region-of-interest vs. whole-brain analysis, statistical thresholds, etc.). Moreover, regarding the context, because ED is a multifaceted phenomenon, broad ecological and psychological factors that depend on subjectivity of the individuals involved in each study may have promoted or influenced different responses at multiple levels, leading to different outcomes in different contexts. In other words, comparability between studies is complicated by heterogeneity between participants (BMI, duration of illness and recovery, etc.), within participants (time of day, hormone levels, etc.) and between studies.

Finally, our inferences from the literature suffer from the same potential biases as multicenter neuroimaging studies. Here, we assumed that scanning site was not a significant source of systematic variance in the observed neural activation patterns.

These limitations explain some of the conflicting results observable in the fMRI studies described above.

### 4.2. Future Directions

The findings reported in this systematic narrative review provide the basis for in-depth considerations of the mechanisms underlying disease development, maintenance and clinical relapse in patients with EDs. We hope that future research will be better detailed and standardized, particularly in terms of comparisons with healthy subjects or in comparisons with other types of psychopathologies, in order to improve theoretical knowledge and clinical outcomes.

## 5. Conclusions

Although we are aware that we still have a long way to go to define precise neuro-functional correlates of eating disorders, we can draw conclusions that summarize the major neurobiological mechanisms discovered in this review of the literature. Anorexia nervosa appears to be associated with general hyperactivity in brain regions involved in both top-down control and emotional areas, such as the amygdala, insula and hypothalamus, as though there are two complementary regulatory strategies. Bulimia nervosa is associated with abnormalities in impulsivity and emotion regulation, resulting in hyperactivity of the insula and striatum. Finally, the neural correlates of binge eating appear to be located in brain structures such as the temporal cortex and striatum, linking this condition to use of dissociative strategies and addictive aspects. The importance of this study is related to tracing the main eating disorders to the substrate of brain activation mechanisms in order to better understand the clinical manifestations and, in addition, to improve the therapeutic treatment of these patients.

## Figures and Tables

**Table 1:** Summary of included studies.

ID	Authors	Date	Eating Disorder	Patients Group (Mean Age or Range)	Healthy Group (Mean Age or Range)
1	Aviram-Friedman et al. [66]	2018	BED	13 BED (18–65)	28 (18–65)
2	Boehm et al. [67]	2018	AN	35 AN (12–29)	35 (12–29)
3	Brooks et al. [68]	2011	BN AN	8 BN (16–50) 42 AN (16–50)	24 (16–50)
4	Brooks et al. [69]	2012	AN	18 AN (16–50)	24 (16–50)
5	Cervantes-Navarrete et al. [70]	2012	AN	5 AN (19–24)	5
6	Dimitropoulos et al. [71]	2012	BED	22 BED (24.8)	16 (24.6)
7	Dodds et al. [72]	2012	BED	26 BED (35.1; 15 M)	/
8	Donnelly et al. [73]	2022	BN BED	14 BN (26.63) 5 BE (26.63)	19 (21.74)
9	Geliebter et al. [74]	2006	BED	10 BED (29–41)	10 (20–24)
10	Gizewski et al. [75]	2010	AN	12 AN (18–52)	12 (21–35)
11	Göller et al. [76]	2022	AN ANrec	31 AN (24.1) 18 ANrec (27.4)	27 (23.6)
12	Holsen et al. [77]	2012	AN, ANrec	12 AN (21.8) 10 ANrec (23.4)	11 (21.6)
13	Horndash et al. [78]	2018	AN young AN adult	15 AN young (16.41) 16 AN adult (26.71)	18 young (15.95) 16 adult (16.88)
14	Joos et al. [79]	2011	AN	11 AN (25)	11 (26)
15	Joos et al. [80]	2011	BN	13 BN (25.2)	13 (27)
16	Kim et al. [81]	2012	AN BN	18 AN (25.2) 20 BN (22.9)	20 (23.3)
17	Lawson et al. [82]	2012	AN ANrec	13 AN (18–28) 9 AN rec (18–28)	13 (18–28)
18	Lee et al. [83]	2017	BN BED	12 BN (23.7) 13 BED (23.6)	14 (23.2)
19	Rothemund et al. [84]	2011	AN	12 AN (24)	12 (26)
20	Sanders et al. [85]	2015	AN ANrec	15 AN (25.6) 15 ANrec (24.3)	15 (25,8)
21	Santel et al. [86]	2006	AN	13 AN (16.1)	10 (16.8)
22	Scaife et al. [87]	2016	AN	12 AN (18–60) 14 ANrec (18–60)	16 (18–60)
23	Schienle et al. [88]	2009	BN BED	14 BN (23.1) 17 BED (26.4)	36 (23.65)
24	Sultson et al. [89]	2016	AN ANrec	14 AN (25.57) 14 ANrec (24.79)	15 (25.8)
25	Uher et al. [90]	2003	AN ANrec	8 AN (25.6) 9 ANrec (26.9)	9 (26.6)
26	Uher et al. [91]	2004	AN BN	16 AN (26.93) 10 BN (29.8)	19 (26.68)
27	Van den Eynde et al. [92]	2013	BN	21 BN (28)	23 (27.3)
28	Wonderlich et al. [93]	2017	BN	16 BN (22.85)	/
29	Young et al. [94]	2020	AN	16 AN (31.4)	20 (26.7)
30	Ziv et al. [95]	2020	AN	18 AN (16.2)	/

Note. AN, anorexia nervosa; ANrec, anorexia nervosa in recovery; BED, binge eating disorder; BN, bulimia nervosa.

## Data Availability

Not applicable.

## Appendix A

**Table A1:** Main results that emerged from the analysis of selected literature.

ID	Paradigm	Stimulus	Conditions	Contrast	Tem-plate	Brain Area	Coordinates
1	Passive viewing	High- and low-energy processed food, neutral non-food	- Food vs. non-food - High vs. low energy - fasting (liquid meal)	BED > nonBED_Food > Non-food_	MNI	Right INS right ACC left PCC right PCC Left MTG LEft CUN Right CUN Right LG Left PoCG Right V2 Right IPL Right dACC	34, −4, 12 8, 8, 44 −38, −64, 18 24,−60, 8 −38, −64, 18 −38, −64, 18 16, −84, 26 24, −60, 8 −66, −22, 22 16,−84, 26 54, −38, 24 8, 8, 44
				BED > nonBED_High > Low_		Left MFG Left SMA Left SFG	−16, −8, 50 −16, −8, 50 −16, −8, 50
2	Passive viewing (“image to eat the following item”)	Food, IAPS, EmoPics	- Food vs. non-food; - Subliminal vs. supraliminal	AN_Food > neutral supra_ (coordinates extrapolated from Appendix A)	MNI	left vSTR left vACC Left SOG Left FFG/PHG	−8, −2, −4 * −8, 46, −2 * −30, −90, 28 −26, −90, 28
3	Passive viewing (“image to eat the food item or to use the non-food item”)	High and low-calorie food, non-food	- Food vs. non-food	AN_Food > Non-food_ _(RAN + BPAN)_ (multiple activations in the cerebellum. Selected one representative)	TAL	Left V2 Right dlPFC Right preCUN Left V2 Left CBM Right CBM Right SMA	−18, −74, −23 47, 7, 30 40, −63, 33 −14, −85, −13 −18, −74, −23 * 25, −63, −20 22, −4, 53
				BN_Food > Non-food_		Right V2 Left dlPFC Right INS Left PrCG	11, −81, −4 −33, 30, 28 −46, 10, −4 −54, −15, 28
				HC_Food > Non-food_		left CBM right STG (INS) right MTG left CN	−4, −63, −20 51, −11, −7 51, −37, 7 −4, 0, 20
				AN > BN_Food > Non-food_ _(RAN + BPAN)_		Right PL Left PCC right PrCG Left ITG	54, −26, −35 −11, −52, 46 43, −4, 43 −36, −56, −17
				BN > AN_Food > Non-food_ _(AN = RAN + BPAN)_		Right CN Right STG(INS) Left SMA Left ITG Left FFG PCC Right ITG Left IPL Left CBM Left PHG Left PCC Right SMA	40, −4, 13 14, 7, 21 −43, 4, 43 −58, −4, −10 −4, −67, −10 0, −41, 13 47, −37, 36 −4, −52, 46 −18, −63, −40 −22, 4, −26 −4, −67, 26 25, 7, 53
				HC > BN_Food > Non-food_		Left STG/INS Right STG/INS Left PCC	54, −26, −7 −51, −15, 0 44, −67, 26
4	Passive viewing (“image to eat the following item or to use the non-food item”)	High and low-calories sweet and savory non-food	- Food vs. non-food	AN_Food > Non-food_ _(RAN + BPAN)_	TAL	Left CBM Left V2 Right dlPFC mPFC Left CBM Right CBM Right ITG	−25, −66, −16 −14, −83, −7 40, 5, 24 0, 42, 40 −4, −56, −27 25, −62, −14 22, −6, −44
				AN > HC_Food > Non-food_		right V2 right V3 right dlPFC	29, −67, 15 29, −75, 20 40, 37, 15
				HC > AN_Food > Non-food_		bil CBM right INS	14, −33, −15 −7, −44, −17 43, −25, 1
				RAN > BPAN_Food > Non-food_		left V2 left PHG left ACC	−11, −78, 17 −18, −32, 2 −4, −18, 32
5	Passive viewing (“image to eat the food presented in the following images”)	High-calorie food, landscapes	- food vs. non-food - fasting state (16 h)	AN > HC_Food > Non-food_	MNI	Left ACC Left mPFC Bil Midbrain	−8, 48, −2 −12, 54, −6 6, −36, −6 −2, −38, −4
6	Perceptual discrimination (“same or different”)	High- and low-calorie food, Objects	- food vs. non-food - high-calorie food vs. non-food - low-calories vs. non-food - premeal(F) vs. postmeal(PF)	BED > HC(F)_Food > Non-food_	TAL	Right aPFC Left aPFC	23, 58, 0 −34, 63, 2
				BED > HC(PF)_Food > Non-food_		Right dlPFC Right OFC Right SFG Right PCC Right TC Right STG Left CBM	0, 53, 21 29, 25, −9 17, 15, 48 18, −46, 0 29, 6, −9 44, 8, −11 −10, −44, −10
				BED > HC(F)_high-calorie > Non-food_		Left aPFC	−33, 63, 0
				BED > HC(PF)_high-calorie > Non-food_		Right PFC Right MFG Right OFC Left ACC Right CN Right HIP	4, 23, 51 2, 47, 37 32, 29, −3 −4, 16, −15 8, 7, 14 27, −35, −2
				BED > HC(F)_low-calorie > Non-food_		right aPFC left aPFC left SFG Right CBM	42, 59, 12 −36, 60, 5 −3, 11, 60 47, −52, −33
				BED > HC(PF)_low-calorie > Non-food_		Left aPFC Right SFG Right dlPFC RIght PCC Left CN Bil aTL Left SMG Right MTG	−16, 59, 3 20, 15, 47 0, 52, 24 21, −48, 3 −2, 22, 3 45, 4, −13 −50, 18, −13 −57, −50, 20 53, −63, 24
				HC > BED_Food > Non-food_		Left dlPFC Left PrCG Left PCC	−29, 28, 35 −46, 0, 7 −23, −26, 44
				HC > BED_high-calorie > Low-calorie_		Left PoCG Left INS Left PHG Bil CBM	−55, −12, 15 −40, −2, 15 −23, −12, −15 45, −50, −34 −16, −65, −19
				HC > BED_high-calorie > Non-food_		Left PFC Left INS Left ACC Left MTG Left STG Left PoCG	−31, 30, 39 −40, −1, 10 −14, −9, 42 −34, −1, −28 −43, −30, 17 −54, −18, 17
7	Passive viewing (“think about how much do you like each image and press a button after every image”)	High and low-calorie food, non-food	- Food vs. non-food; - high-calorie and low-calorie, - fasting (15 h)	BED_Food > non- food_	MNI (ROI)	AMG CN NAc PUT	All left and right ROIs were combined to form single bilateral structures
				BED_High-calorie > low-calorie_		AMG INS NAc PUT	
8	A Rapid Serial Visual Presentation (RSVP) task (“tap your index finger on the buzzers if you saw the same image twice in a row”).	High and low-calorie food, sweet and savory food, non-food, high- and low-energy food images associated to emotions (disgust, fear, happy)	- Low energy (disgust, fear, happy) vs. neutral - High energy (disgust, fear happy) vs. neutral	HC > BEG _Low energy disgust> neutral_ BEG(BN + BED)	MNI	Right CBM Left FFG right CBM right PrCG right CUN left IFG left MFG left PoCG Left PHG	20, −36, −22 −34, −68, −8 8, −65, −10 38, −2, 30 10, −88, 22 −44, 40, −6 −16, −6, 48 −10, −42, 68 38, −12, −22
				BEG > HC_Low energy fear > neutral_		left PCC Right CBM Right PoCG Right MOG Left PhG	−30, −70, 10 14, −46, −8 46, −22, 24 38, −70, 10 −34, −32, −26
				HC > BEG_Low energy fear > neutral_		Right SFG Right MFG Left CBM Right IFG	4, 4, 64 32, 50, 26 −22, −82, −38 50, 22, −2
				BEG > HC_Low energy happy> neutral_		left preCUN	−28, −68, 28
				HC > BEG_High energy disgust > neutral_		right PCC left LG Left MOG right MTG	10, −58, 4 −12, −60, −2 −40, −74, 10 42, 4, −34
				HC > BEG_High energy fear > neutral_		right SFG right IFG	10, 2, 66 24, 36, −8
				BEG > HC_High energy happy > neutral_		left ACC	−2, 2, −6
9	Passive viewing (“attend to the stimuli and queried after each run—hunger ratings and desire to eat”)	Visual and auditory food stimuli, binge food (desserts and high fat salty snacks) non-binge food(fruits and vegetable), Objects	- Binge food stimuli vs. non-binge food stimuli - binge eater (BE) vs. non binge eater (noBE)	BED (BE)_Binge food > Non-binge food_	TAL/BA	bil PrCG bil IFG left LG Left FG	BA (44) BA (45, 46, 57) BA (17, 18) BA (18, 19)
				HC(noBE)_Binge food > Non binge food_		Left IOG Right LG left mOG left ITG	BA (18, 19) BA (17, 18) BA (19) BA (21, 39)
				BED(BE)_Non binge food > Binge food_		Right IFG Right FFG	BA (44) BA (18, 19, 37)
				HC(noBE)_Non binge food > Binge food_		right LG left MOG Left MTG	BA (17, 18) BA (19) BA (21, 39)
10	Passive view (“pay attention to the picture”)	high-calorie food, IAPS,	- hunger (H) [6 h] vs. satiated (S) - high-calories food vs. non-food	AN(H)_high-calories vs. non-food_	TAL	right CUN left IOC left IPL left INS right MCC left PrCG LEft Thal Left AMG Right AMG right OFC	16, −100, −2 −38, −94, −2 −44, −40, 58 −34, 8, 10 6,n8, 48 −58, −12, 30 −4, −20, −2 38, −20, −10 −32, −14, −12 46, 24, −16
				HC(H)_high-calories vs. non-food_		Right IOC left IOC left SPL right SPL left ACC left INS Left AMG Right AMG	38, −70, −8 −40, −72, −14 −22, −60, 56 26, −72, 58 −2, 34, 18 −38, −6, 2 −22, −10, −16 30, 0, −22
				AN(S)_high-calories vs. non-food_		right CUN Left IOC Right SPL Right OFC	24, −90, −8 −44, −82, −8 28, −64, 54 −44, 22, −22
				HC(S)_high-calories vs. non-food_		right CUN left IOC right SPL right PFC Left OFC Left AMG	22, −100, 2 −36, −88, −4 26, −62, 56 48, 32, 6 −28, 50, −14 −24, −8, −20
11	Passive viewing (“Look at each picture and think how hungry it makes you feel and whether you would like to eat the food or not”)	Food, Objects	- food vs. non-food	AN_food > non-food_	MNI	left MOG right OG right LG Left IOG right INS left IOG bil SFG bil pMFC left SFG left MFG left MCC left PreCUN right SMG right PoCG	−21, −97, 8 33, −73, −7 15, −88, −4 −30, −76, −4 0, −31, 35 −12, 20, 65 −18, 50, 35 15, 38, 50 −6, 53, 38 9, 17, 68 0, 59, 23 −9, 56, −7 −9, −7, 32 −6, −52, 20 60, −16, 29 −60, −16, 26
				ANREC_food > non-food_		bil INS left SOrbG left SFG Tlal	−36, −4, 11 39, −1, 2 −21, 35, −13 39, −1, 2 0, −7, 2
				HC_food > non-food_		right V1 left SFG left SOG left SPL left INS right SMG	18, −94, 5 −15, 47, 44 −18, −91, 2 −24, −76, 47 −36, −7, 11 −60, −16, 32
				AN > ANREC_food > non-food_		left FG left V1 left MOG	−33, −58, 11 −21, −58, 8 −30, −73, 5
12	Passive viewing (“look at each image and press a button when pictures change”)	high and low-calorie food, objects	- high-calorie food vs. non-food - fasting(F)[12 h] vs. postmeal (PF)	HC > AN(F)_high-calorie food> non-food_	MNI	Left HYP Left AMG Left HIP Right OFC Bil INS	−3, −7, −5 −21, −10, −11 −9, −40, 1 36, 23, −11 33, 8, 4 −30, 17, 7
				HC > ANrec(F)_high-calorie food> non-food_		Bil HYP Left AMG Right INS	9, −7, −5 −6, −10, −5 −24, −10, −11 39, 26, −8
				HC > AN(PF)_high-calorie food vs. non-food_		Left AMG Left INS	−30, −1, −20 −39, −7, 4
				AN > ANrec(PF)_high-calorie food vs. non-food_		right AMG	15, −1, −17
				ANrec > AN(PF)_high-calorie food vs. non-food_		Bil INS	36, −10, 13 −39, −7, 4
13	Appetite ratings (“look at each picture and rate its valence via button presses”)	Food, IAPS (negative, positive neutral)	- High-calorie vs. low-calorie food - fasting (1.5 h) - adult vs. adolescents	AN > HC_adult high-calorie_	TAL	Bil CBM	28, −70, −24 −18, −70, −24
				AN > HC_adolescents high-calorie_		Right IFG Right mPFC Left INS	22, 28, −2 24, 39, −14 −27, 26, 15
				AN > HC_adult low-calorie_		Bil CBM	28, −70, −24 −41, −68, −21
				AN > HC_adolescents low-calorie_		Left CBM Right mPFG Right IPL	−24, −75, −17 24, 39, −14 53, −43, 37
				Adult > Adolescents_high-calorie_		Left SPL Right CBM	−2, −68, 56 36, −74, −16
				Adolescents > Adults_low-calorie_		Bil ACC Bil SFL Left CBM	8, 35, 13 −3, 33, 24 5, 52, 24 −11, 51, 22 −27, −71, −15
14	Passive view (“Look at each picture and think how hungry it makes you feel and whether you would like to eat the food or not”)	Food, objects, Emotion	- food vs. non-food - Savory and sweet food and Non-food	HC_Food > Non-food_	TAL	Left ACC left MFG left SFL bil INS	−6, 41, 5 −23, 33, 36 −11, 24, 51 34, −1, 11 −34, −7, 11
				AN_Food > Non-food_		right SFL right ACC left MFL AMG left MCC left PreCUN	14, 50, 10 5, 35, 0 −23, 32, 49 29, −5, −6 −2, −14, 31 −5, −52, 22
				AN > HC_Food > Non-food_		right AMG	29, −5, −6
				HC > AN_Food > Non-food_		right pMCC	9, −33, 47
15	Passive view (“Look at each picture and think how hungry it makes you feel and whether you would like to eat the food or not”)	Food, objects, Emotion	- food vs. non-food - Savory and sweet food and Non-food	HC > BN_Food > Non-food_	MNI	Right ACC Right MCC Right mTL	15, 48,24 −9, −18, 48 45, 12, −27
16	Passive view (“imagine tasting the food items or using the non-food items, press a button every time a picture change”)	High-calorie food, Objects	- high-calorie food vs. non-food - fasting (6 h)	AN_Food > Non-food_	MNI	Bil IFG right SFG left ACC left aINS left PreCUN left CUN bil CBM	−51, 15, 4 59, 9, 18 15,7,60 −3, 21, 40 −35, 11, −4 −13, −47, 42 −15, −83, 8 17, −75, −20 −1, −69, −28
				BN_Food > Non-food_		Left aINS left CUN bil CBM	−35, 23, 6 3, −75, 6 41, −71, −24 −39, −67, −22
				HC_Food > Non-food_		left MFG left CUN left LG Right CBM	−45, 27, 26 −5, −93, 10 −1, −85, −6 −37, −65, −20
				AN > HC_Food > Non-food_		right IFG bil SFG left ACC Right CBM	59, 9, 14 13, 7, 58 −7, −1, 46 −7, −35, −26 13, −75, −16
				HC > AN_Food > Non-food_		Right IPL	49, −31, 48
				BN > HC_Food > Non-food_		right MFG right CBM	41, 21, 28 5, −41, −8
				HC > BN_Food > Non-food_		Right PoCG left IPL	11, −41, 66 −27, −59, 42
				AN > BN_Food > Non-food_		bil ACC	1, 19, 42 −11, 17, 32
				BN > AN_Food > Non-food_		right MTG	53, −63, 12
17	Passive viewing	high- and low-calorie food, objects	- High-calorie, low-calorie, non-food - fasting (F) vs. postmeal (PF)	AN > HC (F)	MNI	left HYP left AMG left HIP right OFC Bil INS	−3, −7, −5 −21, −10, −11 −9, −40, 1 36, 23, −11 33, 8, 4 −30, 17, 7
				ANrec > HC (F)		Bil HYP left AMG right INS	9, −7, −5 −6, −10, −5 −24, −10, −11 39, 26, −8
				AN > HC (PF)		left AMG left INS	−30, −1, −20 −33, 5, −5
				ANrec > HC (PF)		right AMG bil INS	15, −1, −17 36, −10, 13 −39, −7, 4
18	Interference from food stimuli on cognitive control	Food, Objects	- Food vs. non-food - fasting (F)[6 h]	ED > HC (BN + BED)	MNI	left dlPFC left OFC left PMC right vSTR right PoCC V2-v3	−18, 44, 48 −24, 20, −8 −42, 8, 54 6, 6, 2 60, 0, 26 16, −92, −4
				BN> HC_Food > Non-food_		Bil dlPFC right dSTR Left PMC Right TPJ Bil V2- v3	20, 44, 48 −16, 34, 58 8, 14, 8 −42, 8, 54 −48, −44, −4 26, −70, 2 −34, −72, 34
				BED > HC_Food > Non-food_		Right vSTR right PoCC	6, 6, 2 60, 0, 28
				BN > BED_Food > Non-food_		bil dlPFC right dSTR left PMC Right TPJ Bil V2-v3	20, 44, 48 −16, 34, 58 6, 14, 10 −40, 8, 52 48, −44, −4 26, −70, 2 −34, −72, 34
				BED > BN_Food > Non-food_		right PoCC left V2-V3	60, 0, 28 −28, −86, 0
19	Passive viewing	Food, utensils, objects	- Low-calorie food, high-calorie food, neutral objects and food utensils	AN_high > neutral_	MNI	Left MOG right IFG bil LG Bil IOG Bil PreCUN right CUN left CUlmen left MTG right SFG left MFG	−48, −76, −4 50, −70, −4 24, −82, −12 −20, −90, −12 40, −76, −10 −30, −88, −14 16, −84, 38 −28, −68, 38 28, −86, 32 −30, −32, −24 −66, −12, −16 16, 56, 22 −46, 50, −12
				AN_low > neutral_		right INS	34, 24, 14
				AN_deactivations_		left MFG right dlPFC	−8, 52, 26 8, 54, 28
				AN_utensil > neutral_		right STG left MFG left claustrum right CC left SupraMG right CG	40, −38, 8 −42, 2, 54 −28, 6, 22 4, 12, 24 −42, −48, 38 4, −40, 40
				HC_utensil > neutral_		right MFG right dlPFC	44, 52, 16 50, 46, 10
				HC_deactivations_		left PreCUN	−22, −70, 32
20	Passive viewing	High- and low-calories, sweet and savory, Utensils	- high-calorie vs. low-calorie, sweet and savory and non-food - fasting [10 h]	AN rec> HC_food_	MNI	right CN right CBM left PoCG left MFG	10, 8, 14 6, −76, −14 −42, −28, 42 −34, 4, 50
				AN > HC_food_		right CBM left MFG	30, −68, −22 −34, 4, 50
				HC > AN_food_		right SFG right PreCUN	14, 40, 50 14, −52, 38
21	Rating stimuli inside fMRI (pleasant, neutral or unpleasant), “keep looking at each picture for as long as it was presented”	High-caloric, sweet and savory food, Objects	- high-caloric sweet and savory food - satiate (S) vs. hungry (H) - fasting [12 h]	AN < HC_S_	TAL	left IPL	−50, −28, 26
				AN < HC_H_		right LG	12, −82, −8
				AN_S_		right IOG right CBM left LG left CBM	27, −91, −6 30, −77, −21 −18, −96, −3 −18, −86, −21
				AN_H_		left CUN right FG	−24, −93, −2 24, −79, −14
				AN_S > H_		right MOG	18, −93, 12
				HC_S_		right CUN right MOG left CUN left IOG	18, −93, 0 30, −90, 10 −12, −99, 0 −12, −91, −8
				HC_H_		right LG Right FG Left LG	21, −91, −3 33, −71, −17 −15, −96, −3
				HC_S > H_		right ACC left OFC left MTG	15, 19, 27 −33, 40, −12 −48, −10, −15
22	Passive viewing (“focus on how much you want each of the different foods, right now”)	High/low-calorie, savory and sweet,	- food vs. baseline - High-calorie vs. low-calorie - fasting [4 h]	HC > AN_food > baseline_	MNI	right PoCG right PreCUN left SPL right PoCG	40, −30, 58 2, −66, 26 −40, −44, 56 48, −24, 58
				AN > HC_high-calorie_ AND HC > AN_lowcalories_		right LFP	28, 64, 0
				HC > AN_lowcalories_		right vmPFC right dlPFC right dmPFC right SMG	8, 52, −20 44, 28, 32 8, 52, 44 62, −48, 16
23	Passive viewing	High- and low-calorie disgusting item, neutral (household articles)	- food vs. non-food- - High-calorie, disgusting and neutral item - fasting [12 h]	BED_Food > Non-food_	MNI	left MOG left MFG Bil INS Bil ACC Bil lOFC Bil mOFC	−15, −96, 0 −24, 30, −21 −36, 6, −15 39, 0, −3 −9, 39, −3 15, 36, 15 −24, 30, −21 21, 27, −21 0, 36, −15 3, 36, −15
				BN_Food > Non-food_		right LG Bil INS Bil ACC Bil lOFC left AMG right vSTR	15, −84, −9 −39, 6, −9 39, 3, −3 −3, 18, 21 6, 18, 21 −27, 33, −12 24, 24, −18 −30, 3, −18 12, 9, −6
				HC_Food > Non-food_		right LG right IFG bil INS Bil ACC Bil lOFC left mOGC Bil AMG left vSTR	9, −93, −6 27, 27, −18 −36, −6, 6 30, 24, −21 −3, 39, 9 3, 33, 12 −27, 33, −15 27, 27, −18 −9, 63, −3 −18, −3, −21 27, 0, −27 −9, 9, −6
				BED > BN_Food > Neutral_		right lOFC right mOFC	36, 33, −12 12, 27, −12
				BED > HC_Food > Neutral_		Bil mOFC	0, 33, −18 6, 36, −12
				BN > BED_Food > Neutral_		Bil ACC right INS	0, 15, 21 3, 15, 24 39, −3, −12
				BN > HC_Food > Neutral_		Bil ACC Bil INS	−6, 21, −6 6, 15, 21 38, −3, −9 −36, −6, −9
24	Passive view (“Imagine eating/using the food/object presented”)	Low- and high-calorie and sweet and savory foods, Objects	- low- and high-calorie, sweet and savory foods and non-food	Average_Food > non-food_ (AN, ANrec, HC—supplementary)	MNI	right MOG left LG left MOG right LG left FFG left CBM right FFG Right AG Bil HPC left MFG vermis left IFG left SupraMG right PoCG left PCL left PreCUN left ACC	30, −84, 6 22, −76, −10 −26, −84, 2 18, −88, −5 −25, −72, −14 −30, −54, −18 34, −56, −18 26, −60, 42 −22, −32, −2 22, −28, −6 −50, 16, 38 2, −40, −6 −46, 12, 30 50, −28, 42 62, −16, 38 −14, −32, 70 −2, −40, 62 −2, 4, 30
25	Passive view (“Look at each picture and think how hungry it makes you feel and whether you would like to eat the food or not”)	savory and sweet, objects, Emotional stimuli (IAPS)	- food vs. non-food - Savory and sweet food and Non-food - aversive stimuli - fasting [3 h]	ANrec_Food > Non-food_	TAL	bil mPFC right lPFC left lPFC right IFG right ACC left CBM	−1, 54, 19 1, 54, 19 36, 53, −7 −48, 44, 6 51, 17, 12 5, 22, 32 −40, −71, −71
				ANrec > HC_Food > Non-food_		bil ACC left CBM	3, 40, 29 −30, −62, −28
				HC > ANrec_Food > Non-food_		left SPL left IPL left V1	−34, −45, 46 −51, −20, 21 −14, −75, 29
				ANrec > AN_Food > Non-food_		left PFC left mPFC right dACC right lPFC left OP left CBM	−5, 58,−15 2, 47, 28 6, 23, 33 50, 19, 12 −34, −60, 39 −34, −62, −22
				AN > ANrec_Food > Non-food_		right LG	5, −61, 6
26	Passive view (“Look at each picture and think how hungry it makes you feel”)	savory and sweet, objects, Emotional stimuli (IAPS)	- Food vs. Non-food - fasting [3 h]	ED > HC_Food > Non-food_	TAL	left vmPFC	−16, 28, −17
				HC > ED_Food > Non-food_		left lPFC left dlPFC left IPL left OC left CBM	−40, 42, 3 −49, 9, 26 −36, −42, 48 −15, −72, 33 −32, −74, −4
				AN > HC_Food > Non-food_		Left vmPFC Right LG	−13, 29, −20 19, −72,−4
				HC > AN_Food > Non-food_		left IPL Left CBM	−33, −47, 44 −32, −73, −20
				BN > HC_Food > Non-food_		Left vmPFC Left LG Bil CBM	−17, 35, −13 −38, −60, −9 2, −54, −20
				HC > BN_Food > Non-food_		left dlPFC left lPFC	−46, 23, 26 −42, 40, 0
				AN > BN_Food > Non-food_		right apicalPFC right lPFC right LG	13, 64, −2 47, 36, −5 17, −72, 1
				BN > AN_Food > Non-food_		right CBM	29, −57, −24
27	Passive viewing (“imagine eating these foods” or “imagine using these tools”)	food, body images, objects	- Food vs. Non-food - own Body vs. others body (no activation enlisted for body images)	BN_Food > Non-food_	TAL	Left SFG Left MFG Left LG	−3.6, 55.6, 29.7 0, 25.9, 36.3 −14.4, −85.2, 0
				BN > HC_Food > Non-food_		Bil CUN	21.7, 77.7, 8.3 −21.7, 77.7, 8.3
				HC_Food > Non-food_		left MFG right CUN	−3.6, 11.1, 42.9 21.7, −77.8, 9.9
28	Distractive task_(related to EMA)_ (“indicate stimulus orientation (landscape vs. portrait) with the button”)	Sweet and savory food, IAPs	- sweet and savory, neutral	BN_food > neutral_	MNI	Bil AMG	-
29	Passive viewing (VAS on anxiety)	high and low-calorie, sweet and savory food, objects	- food vs. non-food - high low-calories, sweet savory	HC_Food > Non-food_	MNI	left CN left V1 Left ACC right CN left Thal left LG	−12, 9, −12 −10, −98, −12 −3, 36, 4 10, 10, −12 −3, −20, 3 −10, −40, −3
				HC_Non-food > Food_		left mOL right CUN left SPL right PreCUN right mTL right IPL	−45, −70, 1 14, −78, 30 −24, −56, 66 12, −52, 54 51, −56, −2 54, −48, 40
				HC > AN_Food > Non-food_		right pgACC	6, 48, 12
30	Passive viewing (“how hungry it makes you feel” VAS on appeals every block)	Food (sweet, processed snack, fast food, meats/fruit), Objects	- Food vs. Non-food	AN_food > non-food_	MNI	Bil V1 Bil LG Bil IOG Right CUN	2, −87, −10
				AN_Non-food > food_		right OG right mTL right CUN right PreCUN right SPL right AG left MTL left STG left OG left SMG	44, −76, 23 −54, −39, 11

Note. ACC, anterior cingulate cortex; AG, angular gyrus; AN, anorexia nervosa; AN_rec_, anorexia nervosa in recovery; BED, binge eating disorder; BN, bulimia nervosa; BOLD, blood-oxygenation-level-dependent; CBM, cerebellum; CN, caudate nucleus; CUN, cuneus; dSTR, dorsal striatum; FFG, fusiform gyrus; HC, healthy controls; HIP, hippocampus; HYP, hypothalamus; IFJ, inferior frontal junction; INS, insula; IOC, inferior occipital cortex; IOG, inferior occipital gyrus; IPL, inferior parietal lobe; ITG, inferior temporal gyrus; LFP, lateral frontal pole; LG, lingual gyrus; MCC, middle cingulate cortex; midOL, middle occipital lobe; MFG, middle frontal gyrus; MOG, middle occipital gyrus; MTG, medial temporal gyrus; midTL, middle temporal lobe; MNI, Montreal Neurological Institute; NAc, nucleus accumbens; OFC, orbitofrontal cortex; OG, occipital gyrus; PCC, posterior cingulate cortex; PFC, prefrontal cortex; pgACC, pregenual anterior cingulate cortex; PHG, parahippocampal gyrus; PL, parietal lobe; PMC, premotor cortex; PoCA, postcentral area; PoCC, postcentral cortex; PoCG, postcentral gyrus; PrCG, precentral gyrus (premotor area); PreCUN, precuneus; Pulv, pulvinar; PUT, putamen; SFL, superior frontal lobe; SFG, superior frontal gyrus; SMA, supplementary motor area; SMG, supramarginal gyrus; SOG, superior occipital gyrus; SOrbG, superior orbital gyrus; SPL, superior parietal lobe; STL, superior temporal lobe; STG, superior temporal gyrus; TAL, Talairach transformation; TC, temporal cortex; Thal, thalamus; TL, temporal lobe; vSTR, ventral striatum; V1, primary visual cortex; V2, secondary visual cortex; V3, tertiary visual cortex.

## References

[1] Gordon R.A. (1990). Anorexia and Bulimia: Anatomy of a Social Epidemic.

[2] Di Fini G., Veglia F. (2019). Life themes and attachment system in the narrative self-construction: Direct and indirect indicators. Front. Psychol..

[3] Köster E.P., Mojet J. (2015). From Mood to Food and from Food to Mood: A Psychological Perspective on the Measurement of Food-Related Emotions in Consumer Research. Food Res. Int..

[4] Dalle Grave R., Centis E., Marzocchi R., El Ghoch M., Marchesini G. (2013). Major Factors for Facilitating Change in Behavioral Strategies to Reduce Obesity. Psychol. Res. Behav. Manag..

[5] Avena N.M., Bocarsly M.E. (2012). Dysregulation of Brain Reward Systems in Eating Disorders: Neurochemical Information from Animal Models of Binge Eating, Bulimia Nervosa, and Anorexia Nervosa. Neuropharmacology.

[6] Kun B., Urbán R., Szabo A., Magi A., Eisinger A., Demetrovics Z. (2022). Emotion Dysregulation Mediates the Relationship between Psychological Distress, Symptoms of Exercise Addiction and Eating Disorders: A Large-Scale Survey among Fitness Center Users. Sport Exerc. Perform. Psychol..

[7] McClure Z., Messer M., Anderson C., Liu C., Linardon J. (2022). Which Dimensions of Emotion Dysregulation Predict the Onset and Persistence of Eating Disorder Behaviours? A Prospective Study. J. Affect. Disord..

[8] Monell E., Clinton D., Birgegård A. (2022). Emotion Dysregulation and Eating Disorder Outcome: Prediction, Change and Contribution of Self-image. Psychol. Psychother. Theory Res. Pract..

[9] American Psychiatric Association (2013). Diagnostic and Statistical Manual of Mental Disorders DSM.

[10] Keski-Rahkonen A., Mustelin L. (2016). Epidemiology of Eating Disorders in Europe: Prevalence, Incidence, Comorbidity, Course, Consequences, and Risk Factors. Curr. Opin. Psychiatry.

[11] Lindvall Dahlgren C., Wisting L., Rø Ø. (2017). Feeding and Eating Disorders in the DSM-5 Era: A Systematic Review of Prevalence Rates in Non-Clinical Male and Female Samples. J. Eat. Disord..

[12] Galmiche M., Déchelotte P., Lambert G., Tavolacci M.P. (2019). Prevalence of Eating Disorders over the 2000–2018 Period: A Systematic Literature Review. Am. J. Clin. Nutr..

[13] Zaccagnino M., Civilotti C., Cussino M., Callerame C., Fernandez I. (2017). EMDR in Anorexia Nervosa: From a Theoretical Framework to the Treatment Guidelines. Eating Disorders—A Paradigm of the Biopsychosocial Model of Illness.

[14] Culbert K.M., Racine S.E., Klump K.L. (2015). Research Review: What We Have Learned about the Causes of Eating Disorders—A Synthesis of Sociocultural, Psychological, and Biological Research. J. Child Psychol. Psychiatry.

[15] Shapiro K.J. (1998). Animal Models of Human Psychology: Critique of Science, Ethics, and Policy.

[16] Overton A., Selway S., Strongman K., Houston M. (2005). Eating Disorders—The Regulation of Positive as Well as Negative Emotion Experience. J. Clin. Psychol. Med. Settings.

[17] Olivo G., Wiemerslage L., Swenne I., Zhukowsky C., Salonen-Ros H., Larsson E.-M., Gaudio S., Brooks S.J., Schiöth H.B. (2017). Limbic-Thalamo-Cortical Projections and Reward-Related Circuitry Integrity Affects Eating Behavior: A Longitudinal DTI Study in Adolescents with Restrictive Eating Disorders. PLoS ONE.

[18] Friederich H.-C., Wu M., Simon J.J., Herzog W. (2013). Neurocircuit Function in Eating Disorders. Int. J. Eat. Disord..

[19] Mercurio A.E., Hong F., Amir C., Tarullo A.R., Samkavitz A., Ashy M., Malley-Morrison K. (2022). Relationships among Childhood Maltreatment, Limbic System Dysfunction, and Eating Disorders in College Women. J. Interpers. Violence.

[20] Farstad S.M., McGeown L.M., von Ranson K.M. (2016). Eating Disorders and Personality, 2004–2016: A Systematic Review and Meta-Analysis. Clin. Psychol. Rev..

[21] Treasure J., Stein D., Maguire S. (2015). Has the Time Come for a Staging Model to Map the Course of Eating Disorders from High Risk to Severe Enduring Illness? An Examination of the Evidence. Early Interv. Psychiatry.

[22] Birmingham C.L., Su J., Hlynsky J.A., Goldner E.M., Gao M. (2005). The Mortality Rate from Anorexia Nervosa. Int. J. Eat. Disord..

[23] Mehler P.S., Watters A., Joiner T., Krantz M.J. (2022). What Accounts for the High Mortality of Anorexia Nervosa?. Int. J. Eat. Disord..

[24] Steinhausen H.-C. (2002). The Outcome of Anorexia Nervosa in the 20th Century. Am. J. Psychiatry.

[25] Qian J., Wu Y., Liu F., Zhu Y., Jin H., Zhang H., Wan Y., Li C., Yu D. (2022). An update on the prevalence of eating disorders in the general population: A systematic review and meta-analysis. Eat Weight Disord..

[26] Van Eeden A.E., van Hoeken D., Hoek H.W. (2021). Incidence, Prevalence and Mortality of Anorexia Nervosa and Bulimia Nervosa. Curr. Opin. Psychiatry.

[27] Hudson J.I., Hiripi E., Pope H.G., Kessler R.C. (2007). The Prevalence and Correlates of Eating Disorders in the National Comorbidity Survey Replication. Biol. Psychiatry.

[28] Allen K.L., Byrne S.M., Oddy W.H., Crosby R.D. (2013). DSM–IV–TR and DSM-5 Eating Disorders in Adolescents: Prevalence, Stability, and Psychosocial Correlates in a Population-Based Sample of Male and Female Adolescents. J. Abnorm. Psychol..

[29] Swanson S.A., Crow S.J., Le Grange D., Swendsen J., Merikangas K.R. (2011). Prevalence and Correlates of Eating Disorders in Adolescents: Results from the National Comorbidity Survey Replication Adolescent Supplement. Arch. Gen. Psychiatry.

[30] Le Grange D., Swanson S.A., Crow S.J., Merikangas K.R. (2012). Eating Disorder Not Otherwise Specified Presentation in the US Population. Int. J. Eat. Disord..

[31] Mangweth-Matzek B., Rupp C.I., Hausmann A., Gusmerotti S., Kemmler G., Biebl W. (2010). Eating Disorders in Men: Current Features and Childhood Factors. Eat. Weight Disord. Anorex. Bulim. Obes..

[32] Raevuori A., Keski-Rahkonen A., Hoek H.W. (2014). A Review of Eating Disorders in Males. Curr. Opin. Psychiatry.

[33] Small D.M., Zatorre R.J., Dagher A., Evans A.C., Jones-Gotman M. (2001). Changes in Brain Activity Related to Eating Chocolate: From Pleasure to Aversion. Brain.

[34] Althubeati S., Avery A., Tench C.R., Lobo D.N., Salter A., Eldeghaidy S. (2022). Mapping Brain Activity of Gut-Brain Signaling to Appetite and Satiety in Healthy Adults: A Systematic Review and Functional Neuroimaging Meta-Analysis. Neurosci. Biobehav. Rev..

[35] Killgore W.D.S., Schwab Z.J., Weber M., Kipman M., DelDonno S.R., Weiner M.R., Rauch S.L. (2013). Daytime Sleepiness Affects Prefrontal Regulation of Food Intake. Neuroimage.

[36] Simmons W.K., Martin A., Barsalou L.W. (2005). Pictures of Appetizing Foods Activate Gustatory Cortices for Taste and Reward. Cereb. Cortex.

[37] O’Doherty J., Rolls E.T., Francis S., Bowtell R., McGlone F., Kobal G., Renner B., Ahne G. (2000). Sensory-Specific Satiety-Related Olfactory Activation of the Human Orbitofrontal Cortex. Neuroreport.

[38] Gearhardt A.N., Yokum S., Orr P.T., Stice E., Corbin W.R., Brownell K.D. (2011). Neural Correlates of Food Addiction. Arch. Gen. Psychiatry.

[39] Azevedo E.P., Ivan V.J., Friedman J.M., Stern S.A. (2022). Higher-Order Inputs Involved in Appetite Control. Biol. Psychiatry.

[40] Kroemer N.B., Krebs L., Kobiella A., Grimm O., Pilhatsch M., Bidlingmaier M., Zimmermann U.S., Smolka M.N. (2013). Fasting Levels of Ghrelin Covary with the Brain Response to Food Pictures. Addict. Biol..

[41] Peters R., White D.J., Scholey A. (2020). Resting State FMRI Reveals Differential Effects of Glucose Administration on Central Appetite Signalling in Young and Old Adults. J. Psychopharmacol..

[42] LaBar K.S., Gitelman D.R., Parrish T.B., Kim Y.H., Nobre A.C., Mesulam M.M. (2001). Hunger Selectively Modulates Corticolimbic Activation to Food Stimuli in Humans. Behav. Neurosci..

[43] Grimm O., Jacob M.J., Kroemer N.B., Krebs L., Vollstädt-Klein S., Kobiella A., Wolfensteller U., Smolka M.N. (2012). The Personality Trait Self-Directedness Predicts the Amygdala’s Reaction to Appetizing Cues in fMRI. Appetite.

[44] Gordon C.M., Dougherty D.D., Rauch S.L., Emans S.J., Grace E., Lamm R., Alpert N.M., Majzoub J.A., Fischman A.J. (2000). Neuroanatomy of Human Appetitive Function: A Positron Emission Tomography Investigation. Int. J. Eat. Disord..

[45] Price A.E., Stutz S.J., Hommel J.D., Anastasio N.C., Cunningham K.A. (2019). Anterior Insula Activity Regulates the Associated Behaviors of High Fat Food Binge Intake and Cue Reactivity in Male Rats. Appetite.

[46] Schur E.A., Kleinhans N.M., Goldberg J., Buchwald D., Schwartz M.W., Maravilla K. (2009). Activation in Brain Energy Regulation and Reward Centers by Food Cues Varies with Choice of Visual Stimulus. Int. J. Obes..

[47] Sewaybricker L.E., Melhorn S.J., Rosenbaum J.L., Askren M.K., Tyagi V., Webb M.F., De Leon M.R.B., Grabowski T.J., Schur E.A. (2021). Reassessing Relationships between Appetite and Adiposity in People at Risk of Obesity: A Twin Study Using FMRI. Physiol. Behav..

[48] Volkow N.D., Wang G.-J., Telang F., Fowler J.S., Thanos P.K., Logan J., Alexoff D., Ding Y.-S., Wong C., Ma Y. (2008). Low Dopamine Striatal D2 Receptors Are Associated with Prefrontal Metabolism in Obese Subjects: Possible Contributing Factors. Neuroimage.

[49] Fulton S. (2010). Appetite and Reward. Front. Neuroendocrinol..

[50] Contreras-Rodriguez O., Burrows T., Pursey K.M., Stanwell P., Parkes L., Soriano-Mas C., Verdejo-Garcia A. (2019). Food Addiction Linked to Changes in Ventral Striatum Functional Connectivity between Fasting and Satiety. Appetite.

[51] García-García I., Kube J., Morys F., Schrimpf A., Kanaan A.S., Gaebler M., Villringer A., Dagher A., Horstmann A., Neumann J. (2020). Liking and Left Amygdala Activity during Food versus Nonfood Processing Are Modulated by Emotional Context. Cogn. Affect. Behav. Neurosci..

[52] Holsen L.M., Zarcone J.R., Thompson T.I., Brooks W.M., Anderson M.F., Ahluwalia J.S., Nollen N.L., Savage C.R. (2005). Neural Mechanisms Underlying Food Motivation in Children and Adolescents. Neuroimage.

[53] Kringelbach M.L. (2004). Food for Thought: Hedonic Experience beyond Homeostasis in the Human Brain. Neuroscience.

[54] Zald D.H. (2003). The Human Amygdala and the Emotional Evaluation of Sensory Stimuli. Brain Res. Rev..

[55] Chen T., Cai W., Ryali S., Supekar K., Menon V. (2016). Distinct Global Brain Dynamics and Spatiotemporal Organization of the Salience Network. PLoS Biol..

[56] Beaver J.D., Lawrence A.D., Van Ditzhuijzen J., Davis M.H., Woods A., Calder A.J. (2006). Individual Differences in Reward Drive Predict Neural Responses to Images of Food. J. Neurosci..

[57] Hare T.A., O’doherty J., Camerer C.F., Schultz W., Rangel A. (2008). Dissociating the Role of the Orbitofrontal Cortex and the Striatum in the Computation of Goal Values and Prediction Errors. J. Neurosci..

[58] Goldstone A.P., de Hernandez C.G.P., Beaver J.D., Muhammed K., Croese C., Bell G., Durighel G., Hughes E., Waldman A.D., Frost G. (2009). Fasting Biases Brain Reward Systems towards High-Calorie Foods. Eur. J. Neurosci..

[59] Davidenko O., Bonny J.-M., Morrot G., Jean B., Claise B., Benmoussa A., Fromentin G., Tomé D., Nadkarni N., Darcel N. (2018). Differences in BOLD Responses in Brain Reward Network Reflect the Tendency to Assimilate a Surprising Flavor Stimulus to an Expected Stimulus. Neuroimage.

[60] Giel K.E., Friederich H.-C., Teufel M., Hautzinger M., Enck P., Zipfel S. (2011). Attentional Processing of Food Pictures in Individuals with Anorexia Nervosa—An Eye-Tracking Study. Biol. Psychiatry.

[61] Livneh Y., Ramesh R.N., Burgess C.R., Levandowski K.M., Madara J.C., Fenselau H., Goldey G.J., Diaz V.E., Jikomes N., Resch J.M. (2017). Homeostatic Circuits Selectively Gate Food Cue Responses in Insular Cortex. Nature.

[62] Suzuki S., Cross L., O’Doherty J.P. (2017). Elucidating the Underlying Components of Food Valuation in the Human Orbitofrontal Cortex. Nat. Neurosci..

[63] Seabrook L.T., Borgland S.L. (2020). The Orbitofrontal Cortex, Food Intake and Obesity. J. Psychiatry Neurosci..

[64] Page M.J., McKenzie J.E., Bossuyt P.M., Boutron I., Hoffmann T.C., Mulrow C.D., Shamseer L., Tetzlaff J.M., Akl E.A., Brennan S.E. (2021). The PRISMA 2020 Statement: An Updated Guideline for Reporting Systematic Reviews. Int. J. Surg..

[65] Moher D., Liberati A., Tetzlaff J., Altman D.G. (2009). PRISMA Group the PRISMA Group Preferred Reporting Items for Systematic Reviews and Meta-Analyses. Prism. Statement BMJ.

[66] Aviram-friedman R., Astbury N., Ochner C.N., Contento I. (2018). Physiology & Behavior Neurobiological Evidence for Attention Bias to Food, Emotional Dysregulation, Disinhibition and de Fi Cient Somatosensory Awareness in Obesity with Binge Eating Disorder. Physiol. Behav..

[67] Boehm I., King J.A., Bernardoni F., Geisler D., Seidel M., Ritschel F., Goschke T., Haynes J.-D., Roessner V., Ehrlich S. (2018). Subliminal and Supraliminal Processing of Reward-Related Stimuli in Anorexia Nervosa. Psychol. Med..

[68] Brooks S.J., ODaly O.G., Uher R., Friederich H.-C., Giampietro V., Brammer M., Williams S.C.R., Schiöth H.B., Treasure J., Campbell I.C. (2011). Differential Neural Responses to Food Images in Women with Bulimia versus Anorexia Nervosa. PLoS ONE.

[69] Brooks S.J., O’Daly O., Uher R., Friederich H.-C., Giampietro V., Brammer M., Williams S.C.R., Schiöth H.B., Treasure J., Campbell I.C. (2012). Thinking about Eating Food Activates Visual Cortex with Reduced Bilateral Cerebellar Activation in Females with Anorexia Nervosa: An fMRI Study. PLoS ONE.

[70] Cervantes-Navarrete J.J., Alcauter-Solórzano S., Miguel-Bueno C., Gonzalez-Olvera J.J., Carrillo-Mezo R., De Lourdes Martínez-Gudiño M., De Jesús Caballero-Romo A. (2012). Neurofunctional Areas Related to Food Appetency in Anorexia Nervosa. J. Psychol. Res..

[71] Dimitropoulos A., Tkach J., Ho A., Kennedy J. (2012). Greater Corticolimbic Activation to High-Calorie Food Cues after Eating in Obese vs. Normal-Weight Adults. Appetite.

[72] Dodds C.M., O’Neill B., Beaver J., Makwana A., Bani M., Merlo-Pich E., Fletcher P.C., Koch A., Bullmore E.T., Nathan P.J. (2012). Effect of the Dopamine D 3 Receptor Antagonist GSK598809 on Brain Responses to Rewarding Food Images in Overweight and Obese Binge Eaters. Appetite.

[73] Donnelly B., Williams M., Touyz S., Madden S., Kohn M., Clark S., Caterson I., Russell J. (2022). Neural Response to Low Energy and High Energy Foods in Bulimia Nervosa and Binge Eating Disorder: A Functional MRI Study. Front. Psychol..

[74] Geliebter A., Ladell T., Logan M., Schweider T., Sharafi M., Hirsch J. (2006). Responsivity to Food Stimuli in Obese and Lean Binge Eaters Using Functional MRI. Appetite.

[75] Gizewski E.R., Rosenberger C., de Greiff A., Moll A., Senf W., Wanke I., Forsting M., Herpertz S. (2010). Influence of Satiety and Subjective Valence Rating on Cerebral Activation Patterns in Response to Visual Stimulation with High-Calorie Stimuli among Restrictive Anorectic and Control Women. Neuropsychobiology.

[76] Göller S., Nickel K., Horster I., Endres D., Zeeck A., Domschke K., Lahmann C., Van Elst L.T., Maier S., Joos A.A.B. (2022). State or Trait: The Neurobiology of Anorexia Nervosa-Contributions of a Functional Magnetic Resonance Imaging Study. J. Eat. Disord..

[77] Holsen L.M., Lawson E.A., Blum J., Ko E., Makris N., Fazeli P.K., Klibanski A., Goldstein J.M. (2012). Food Motivation Circuitry Hypoactivation Related to Hedonic and Nonhedonic Aspects of Hunger and Satiety in Women with Active Anorexia Nervosa and Weight-Restored Women with Anorexia Nervosa. J. Psychiatry Neurosci..

[78] Horndasch S., Roesch J., Forster C., Doerfler A., Lindsiepe S., Heinrich H., Graap H., Moll G.H., Kratz O. (2018). Neural Processing of Food and Emotional Stimuli in Adolescent and Adult Anorexia Nervosa Patients. PLoS ONE.

[79] Joos A.A.B., Saum B., Tebartz L., Elst V., Perlov E., Glauche V., Hartmann A., Freyer T., Tüscher O., Zeeck A. (2011). Psychiatry Research: Neuroimaging Amygdala Hyperreactivity in Restrictive Anorexia Nervosa. Psychiatry Res. Neuroimaging.

[80] Joos A.A.B., Saum B., Zeeck A., Perlov E., Glauche V., Hartmann A., Freyer T., Sandholz A., Unterbrink T., Van Elst L.T. (2011). Frontocingular Dysfunction in Bulimia Nervosa When Confronted with Disease-Specific Stimuli. Eur. Eat. Disord. Rev..

[81] Kim K.R., Ku J., Lee J.-H., Lee H., Jung Y.-C. (2012). Functional and Effective Connectivity of Anterior Insula in Anorexia Nervosa and Bulimia Nervosa. Neurosci. Lett..

[82] Lawson E.A., Holsen L.M., Santin M., Meenaghan E., Eddy K.T., Becker A.E., Herzog D.B., Goldstein J.M., Klibanski A. (2012). Oxytocin Secretion Is Associated with Severity of Disordered Eating Psychopathology and Insular Cortex Hypoactivation in Anorexia Nervosa. J. Clin. Endocrinol. Metab..

[83] Lee J.E., Namkoong K., Jung Y.-C. (2017). Impaired Prefrontal Cognitive Control over Interference by Food Images in Binge-Eating Disorder and Bulimia Nervosa. Neurosci. Lett..

[84] Rothemund Y., Buchwald C., Georgiewa P., Bohner G., Bauknecht H.-C., Ballmaier M., Klapp B.F., Klingebiel R. (2011). Compulsivity Predicts Fronto Striatal Activation in Severely Anorectic Individuals. Neuroscience.

[85] Neuroscience B., Sanders N., Smeets P.A.M., Van Elburg A.A., Danner U.N., Van Meer F., Hoek H.W., Adan R.A.H. (2015). Altered Food-Cue Processing in Chronically Ill and Recovered Women with Anorexia Nervosa. Front. Behav. Neurosci..

[86] Santel S., Baving L., Krauel K., Muente T.F., Rotte M. (2006). Hunger and Satiety in Anorexia Nervosa: FMRI during Cognitive Processing of Food Pictures. Brain Res..

[87] Scaife J.C., Godier L.R., Reinecke A., Harmer C.J., Park R.J. (2016). Differential Activation of the Frontal Pole to High vs Low Calorie Foods: The Neural Basis of Food Preference in Anorexia Nervosa?. Psychiatry Res..

[88] Schienle A., Schaefer A., Hermann A., Vaitl D. (2009). Binge-Eating Disorder: Reward Sensitivity and Brain Activation to Images of Food. Biol. Psychiatry.

[89] Sultson H., Van Meer F., Sanders N., Van Elburg A.A., Danner U.N., Hoek H.W., Adan R.A.H., Smeets P.A.M. (2016). Psychiatry Research: Neuroimaging Associations between Neural Correlates of Visual Stimulus Processing and Set-Shifting in Ill and Recovered Women with Anorexia Nervosa. Psychiatry Res. Neuroimaging.

[90] Uher R., Brammer M.J., Murphy T., Campbell I.C., Ng V.W., Williams S.C.R., Treasure J. (2003). Recovery and Chronicity in Anorexia Nervosa: Brain Activity Associated with Differential Outcomes. Biol. Psychiatry.

[91] Uher R., Murphy T., Brammer M.J., Dalgleish T., Phillips M.L., Ng V.W., Andrew C.M., Williams S.C., Campbell I.C., Treasure J. (2004). Medial Prefrontal Cortex Activity Associated with Symptom Provocation in Eating Disorders. Am. J. Psychiatry.

[92] Van Den Eynde F., Giampietro V., Simmons A., Uher R., Andrew C.M., Harvey P., Campbell I.C., Schmidt U. (2013). Brain Responses to Body Image Stimuli but Not Food Are Altered in Women with Bulimia Nervosa. BMC Psychiatry.

[93] Wonderlich J.A., Breithaupt L.E., Crosby R.D., Thompson J.C., Engel S.G., Fischer S. (2017). The Relation between Craving and Binge Eating: Integrating Neuroimaging and Ecological Momentary Assessment. Appetite.

[94] Young K.S., Rennalls S.J., Leppanen J., Mataix-cols D., Simmons A., Suda M., Campbell I.C., Daly O.O., Cardi V. (2020). Journal of A Ff Ective Disorders Exposure to Food in Anorexia Nervosa and Brain Correlates of Food-Related Anxiety: Fi Ndings from a Pilot Study. J. Affect. Disord..

[95] Ziv A., O’Donnell J.M., Ofei-Tenkorang N., Meisman A.R., Nash J.K., Mitan L.P., DiFrancesco M., Altaye M., Gordon C.M. (2020). Correlation of Functional Magnetic Resonance Imaging Response to Visual Food Stimuli With Clinical Measures in Adolescents With Restrictive Eating Disorders. J. Adolesc. Health.

[96] Gao Q., Horvath T.L. (2007). Neurobiology of Feeding and Energy Expenditure. Annu. Rev. Neurosci..

[97] Killgore W.D.S., Young A.D., Femia L.A., Bogorodzki P., Rogowska J., Yurgelun-Todd D.A. (2003). Cortical and Limbic Activation during Viewing of High- versus Low-Calorie Foods. Neuroimage.

[98] Lappalainen R., Sjödén P.O. (1992). A Functional Analysis of Food Habits. Scand. J. Nutr..

[99] Rolls B.J. (1999). Do Chemosensory Changes Influence Food Intake in the Elderly?. Physiol. Behav..

[100] Holland P.C., Petrovich G.D. (2005). A Neural Systems Analysis of the Potentiation of Feeding by Conditioned Stimuli. Physiol. Behav..

[101] Cowdrey F.A., Park R.J., Harmer C.J., McCabe C. (2011). Increased Neural Processing of Rewarding and Aversive Food Stimuli in Recovered Anorexia Nervosa. Biol. Psychiatry.

[102] Wagner A., Barbarich-Marsteller N.C., Frank G.K., Bailer U.F., Wonderlich S.A., Crosby R.D., Henry S.E., Vogel V., Plotnicov K., McConaha C. (2006). Personality Traits after Recovery from Eating Disorders: Do Subtypes Differ?. Int. J. Eat. Disord..

[103] Führer D., Zysset S., Stumvoll M. (2008). Brain Activity in Hunger and Satiety: An Exploratory Visually Stimulated FMRI Study. Obesity.

[104] Herbert B.M., Pollatos O. (2018). The Relevance of Interoception for Eating Behavior and Eating Disorders. Interoceptive Mind Homeost. Aware..

[105] Kim B. (2018). The Role of the Dorsal Striatum in Choice Impulsivity. Ann. N. Y. Acad. Sci..

[106] Simon J.J., Stopyra M.A., Friederich H.-C. (2019). Neural Processing of Disorder-Related Stimuli in Patients with Anorexia Nervosa: A Narrative Review of Brain Imaging Studies. J. Clin. Med..

[107] Epstein J., Wiseman C.V., Sunday S.R., Klapper F., Alkalay L., Halmi K.A. (2001). Neurocognitive Evidence Favors “Top down” over “Bottom up” Mechanisms in the Pathogenesis of Body Size Distortions in Anorexia Nervosa. Eat. Weight Disord. Anorex. Bulim. Obes..

[108] Civilotti C., Franceschinis M., Gandino G., Veglia F., Anselmetti S., Bertelli S., Agostino A.D., Redaelli C.A., Del Giudice R., Giampaolo R. (2022). State of Mind Assessment in Relation to Adult Attachment and Text Analysis of Adult Attachment Interviews in a Sample of Patients with Anorexia Nervosa. Eur. J. Investig. Health Psychol. Educ..

[109] Carlén M. (2017). What Constitutes the Prefrontal Cortex?. Science.

[110] Parto Dezfouli M., Zarei M., Constantinidis C., Daliri M.R. (2021). Task-Specific Modulation of PFC Activity for Matching-Rule Governed Decision-Making. Brain Struct. Funct..

[111] Van Gaal S., Lamme V.A.F. (2012). Unconscious High-Level Information Processing: Implication for Neurobiological Theories of Consciousness. Neuroscience.

[112] Phillips A.G., Vacca G., Ahn S. (2008). A Top-down Perspective on Dopamine, Motivation and Memory. Pharmacol. Biochem. Behav..

[113] Sarter M., Givens B., Bruno J.P. (2001). The Cognitive Neuroscience of Sustained Attention: Where Top-down Meets Bottom-Up. Brain Res. Rev..

[114] De Kloet E.R., de Kloet S.F., de Kloet C.S., de Kloet A.D. (2019). Top-down and Bottom-up Control of Stress-coping. J. Neuroendocrinol..

[115] Brand-Gothelf A., Leor S., Apter A., Fennig S. (2014). The Impact of Comorbid Depressive and Anxiety Disorders on Severity of Anorexia Nervosa in Adolescent Girls. J. Nerv. Ment. Dis..

[116] Mitchell J.E., Crow S. (2006). Medical Complications of Anorexia Nervosa and Bulimia Nervosa. Curr. Opin. Psychiatry.

[117] Kaye W.H., Bulik C.M., Thornton L., Barbarich N., Masters K., Group P.F.C. (2004). Comorbidity of Anxiety Disorders with Anorexia and Bulimia Nervosa. Am. J. Psychiatry.

[118] Bruch H. (2001). The Golden Cage: The Enigma of Anorexia Nervosa.

[119] Jäncke L., Mirzazade S., Shah N.J. (1999). Attention Modulates Activity in the Primary and the Secondary Auditory Cortex: A Functional Magnetic Resonance Imaging Study in Human Subjects. Neurosci. Lett..

[120] Gazzaley A., Cooney J.W., Rissman J., D’esposito M. (2005). Top-down Suppression Deficit Underlies Working Memory Impairment in Normal Aging. Nat. Neurosci..

[121] Balbo M., Zaccagnino M., Cussino M., Civilotti C. (2017). Eye Movement Desensitization and Reprocessing (EMDR) and Eating Disorders: A Systematic Review. Clin. Neuropsychiatry.

[122] Civilotti C., Cussino M., Callerame C., Fernandez I., Zaccagnino M. (2019). Changing the Adult State of Mind With Respect to Attachment: An Exploratory Study of the Role of EMDR Psychotherapy. J. EMDR Pract. Res..

[123] Slof-Op’t Landt M.C.T., Dingemans A.E., Giltay E.J. (2022). Eating Disorder Psychopathology Dimensions Based on Individual Co-Occurrence Patterns of Symptoms over Time: A Dynamic Time Warp Analysis in a Large Naturalistic Patient Cohort. Eat. Weight Disord. Anorex. Bulim. Obes..

[124] Newton J.R., Freeman C.P., Munro J. (1993). Impulsivity and Dyscontrol in Bulimia Nervosa: Is Impulsivity an Independent Phenomenon or a Marker of Severity?. Acta Psychiatr. Scand..

[125] Fischer S., Smith G.T., Anderson K.G. (2003). Clarifying the Role of Impulsivity in Bulimia Nervosa. Int. J. Eat. Disord..

[126] Mallorquí-Bagué N., Testa G., Lozano-Madrid M., Vintró-Alcaraz C., Sánchez I., Riesco N., Granero R., Perales J.C., Navas J.F., Megías-Robles A. (2020). Emotional and Non-emotional Facets of Impulsivity in Eating Disorders: From Anorexia Nervosa to Bulimic Spectrum Disorders. Eur. Eat. Disord. Rev..

[127] Bagnis A., Celeghin A., Diano M., Mendez C.A., Spadaro G., Mosso C.O., Avenanti A., Tamietto M. (2020). Functional Neuroanatomy of Racial Categorization from Visual Perception: A Meta-Analytic Study. Neuroimage.

[128] Kim J.-Y., Chun J.-W., Park C.-H., Cho H., Choi J., Yang S., Ahn K.-J., Kim D.J. (2019). The Correlation between the Frontostriatal Network and Impulsivity in Internet Gaming Disorder. Sci. Rep..

[129] Reichl D., Enewoldsen N., Weisel K.K., Saur S., Fuhrmann L., Lang C., Berking M., Zink M., Ahnert A., Falkai P. (2022). Lower Emotion Regulation Competencies Mediate the Association between Impulsivity and Craving during Alcohol Withdrawal Treatment. Subst. Use Misuse.

[130] Sun T., Song Z., Tian Y., Tian W., Zhu C., Ji G., Luo Y., Chen S., Wang L., Mao Y. (2019). Basolateral Amygdala Input to the Medial Prefrontal Cortex Controls Obsessive-Compulsive Disorder-like Checking Behavior. Proc. Natl. Acad. Sci. USA.

[131] Engel S.G., Corneliussen S.J., Wonderlich S.A., Crosby R.D., Le Grange D., Crow S., Klein M., Bardone-Cone A., Peterson C., Joiner T. (2005). Impulsivity and Compulsivity in Bulimia Nervosa. Int. J. Eat. Disord..

[132] Howard M., Gregertsen E.C., Hindocha C., Serpell L. (2020). Impulsivity and Compulsivity in Anorexia and Bulimia Nervosa: A Systematic Review. Psychiatry Res..

[133] Von Ranson K.M., Kaye W.H., Weltzin T.E., Rao R., Matsunaga H. (1999). Obsessive-Compulsive Disorder Symptoms before and after Recovery from Bulimia Nervosa. Am. J. Psychiatry.

[134] Vanzhula I.A., Kinkel-Ram S.S., Levinson C.A. (2021). Perfectionism and Difficulty Controlling Thoughts Bridge Eating Disorder and Obsessive-Compulsive Disorder Symptoms: A Network Analysis. J. Affect. Disord..

[135] Leslie M., Lambert E., Treasure J. (2019). Towards a Translational Approach to Food Addiction: Implications for Bulimia Nervosa. Curr. Addict. Rep..

[136] Fauconnier M., Rousselet M., Brunault P., Thiabaud E., Lambert S., Rocher B., Challet-Bouju G., Grall-Bronnec M. (2020). Food Addiction among Female Patients Seeking Treatment for an Eating Disorder: Prevalence and Associated Factors. Nutrients.

[137] Hail L., Le Grange D. (2018). Bulimia Nervosa in Adolescents: Prevalence and Treatment Challenges. Adolesc. Health Med. Ther..

[138] Smith D.G., Robbins T.W. (2013). The Neurobiological Underpinnings of Obesity and Binge Eating: A Rationale for Adopting the Food Addiction Model. Biol. Psychiatry.

[139] Jáuregui-Lobera I., Montes-Martínez M. (2020). Emotional Eating and Obesity. Psychosomatic Medicine.

[140] Engelberg M.J., Steiger H., Gauvin L., Wonderlich S.A. (2007). Binge Antecedents in Bulimic Syndromes: An Examination of Dissociation and Negative Affect. Int. J. Eat. Disord..

[141] La Mela C., Maglietta M., Castellini G., Amoroso L., Lucarelli S. (2010). Dissociation in Eating Disorders: Relationship between Dissociative Experiences and Binge-Eating Episodes. Compr. Psychiatry.

[142] McShane J.M., Zirkel S. (2008). Dissociation in the Binge–Purge Cycle of Bulimia Nervosa. J. Trauma Dissociation.

[143] Hopper J.W., Frewen P.A., van der Kolk B.A., Lanius R.A. (2007). Neural Correlates of Reexperiencing, Avoidance, and Dissociation in PTSD: Symptom Dimensions and Emotion Dysregulation in Responses to Script-driven Trauma Imagery. J. Trauma. Stress.

[144] Elzinga B.M., Ardon A.M., Heijnis M.K., De Ruiter M.B., Van Dyck R., Veltman D.J. (2007). Neural Correlates of Enhanced Working-Memory Performance in Dissociative Disorder: A Functional MRI Study. Psychol. Med..

[145] Roydeva M.I., Reinders A.A.T.S. (2021). Biomarkers of Pathological Dissociation: A Systematic Review. Neurosci. Biobehav. Rev..

[146] Lindgren E., Gray K., Miller G., Tyler R., Wiers C.E., Volkow N.D., Wang G.-J. (2018). Food Addiction: A Common Neurobiological Mechanism with Drug Abuse. Front. Biosci..

[147] Nakamura Y., Ikuta T. (2017). Caudate-Precuneus Functional Connectivity Is Associated with Obesity Preventive Eating Tendency. Brain Connect..

[148] Fuller-Tyszkiewicz M., Mussap A.J. (2008). The Relationship between Dissociation and Binge Eating. J. Trauma Dissociation.

